# Fungicide resistance profiles of *Alternaria* spp. associated with fruit rot of blueberry in Georgia, USA

**DOI:** 10.3389/fpls.2025.1524586

**Published:** 2025-02-27

**Authors:** Mirza Ashikul Beg, Md. Aktaruzzaman, Kippy J. Lewis, Jonathan E. Oliver

**Affiliations:** Department of Plant Pathology, University of Georgia, Tifton, GA, United States

**Keywords:** *Alternaria* spp., blueberry, *Vaccinium* spp., fruit rot, fungicide resistance, resistance

## Abstract

Georgia blueberry growers experience significant losses annually due to fruit rots including Alternaria rot caused by *Alternaria* spp. Fungicide applications from bloom through harvest are typically recommended for management of fruit rots, however fungicide resistance development has the potential to complicate management activities by reducing fungicide efficacy. To evaluate fungicide resistance issues in Georgia, 46 isolates of *Alternaria* spp. from ripe blueberry fruit from four major blueberry-producing counties were collected and identified by morphological and molecular features. The majority of the isolates were *Alternaria alternata* (n=43) but also included *Alternaria tenuissima* (n=1), *Alternaria dumosa* (n=1), and *Alternaria limoniasperae* (n=1). All isolates were assessed for resistance to fungicides which included fludioxonil, fluazinam, metconazole, cyprodinil, pydiflumetofen, boscalid, and pyraclostrobin. For all tested fungicides, with the exception of pyraclostrobin, a mycelial growth inhibition assay was used to determine the EC_50_ values. For pyraclostrobin, a spore germination assay was used. EC_50_ value ranges of *A. alternata* for fludioxonil, fluazinam, cyprodinil, metconazole, pydiflumetofen, boscalid, and pyraclostrobin were 0.037 to 0.234 µg/mL, 0.025 to 0.125 µg/mL, 0.015 to 0.404 µg/mL, 0.125 to 5.729 µg/mL, 0.008 to 1.114 µg/mL, 0.551 to >100 µg/mL, and 0.04 to >100 µg/mL, respectively. These EC_50_ values suggest that all tested *Alternaria* spp. isolates were sensitive to fludioxonil, fluazinam, metconazole, and cyprodinil. However, 12 *Alternaria* spp. isolates showed reduced sensitivity to pydiflumetofen, 21 were resistant to boscalid and 10 were resistant to pyraclostrobin. Among these resistant isolates, 6 were resistant to both of the two latter fungicides. Sequencing portions of the *sdhB*, *sdhC* and *sdhD* genes from boscalid-resistant isolates and the cytochrome b gene from pyraclostrobin-resistant isolates revealed the presence of known resistance mutations in resistant isolates - including H134Q or G79R mutations in the *sdhC* gene or H134R mutations in the *sdhD* gene of some, but not all, boscalid-resistant isolates, and the presence of the G143A mutation in pyraclostrobin-resistant isolates. Our findings indicate that resistance to boscalid and pyraclostrobin is present in *Alternaria* spp. from Georgia blueberries and suggest that growers utilizing these fungicides in some Georgia locations may experience Alternaria fruit rot control failures.

## 1 Introduction

Blueberry is a very popular fruit in the United States, in high demand not only for its taste but also due to the health benefits that it provides. High levels of antioxidants are considered to help improve cognitive performance and reduce the risk of cardiovascular disease and aging-related damage (Hein et al., 2019; Wood et al., 2019). Globally, blueberry production increased from 419,050 metric tons to nearly 1,934,400 metric tons between 2009 to 2021, and production is forecast to reach 3,000,000 metric tons by 2025 (IBO, 2022). Georgia ranks first in the U.S. with 20,600 harvested acres (NASS, 2022), and blueberries have a farmgate value estimated of $300 million, accounting for over 41% of the total value of fruits and nuts produced in Georgia (UGA, 2021).

The small size and soft outer skin of blueberry fruit make them especially vulnerable to pathogens. Several studies have shown that postharvest degradation of rabbiteye (*Vaccinium virgatum*) and southern highbush (*Vaccinium corymbosum* interspecific hybrids) blueberries is a significant barrier to production (Barrau et al., 2006), and major losses from blueberry fruit rots can occur both in the field and after harvest during postharvest handling and storage (Neugebauer et al., 2024). The primary postharvest fruit rots of blueberries are caused by fungi, with *Botrytis cinerea* (gray mold), *Alternaria* spp. (Alternaria fruit rot), and *Colletotrichum* spp. (anthracnose fruit rot) as the major contributors (Bell et al., 2021; Neugebauer et al., 2024). Though there are many different species of *Alternaria* that cause postharvest diseases in different fruits, *A. alternata, A. tenuissima*, and *A. arborescens* are the most common species that cause Alternaria rot in blueberries (Neugebauer et al., 2024). A survey conducted in California on Alternaria rot on blueberries showed that 62% of the isolates were *A. alternata*, 33% were *A. arborescens* and 5% were *A. tenuissima* (Zhu and Xiao, 2015). These pathogens are very important because they cause rots not only in blueberries but in many other diverse fruits and vegetables including apple, pepper, mandarin, and pomegranates (Cabral et al., 2016; Luo et al., 2017; Elfar et al., 2018; Wang et al., 2021).

Infection by *Alternaria* spp. can occur as early as bloom, but infections typically remain latent and become apparent when fruit ripens (Neugebauer et al., 2024). Initially the ripe fruit shrivels or flattens. The damaged part later gets covered with a greenish mass of mycelium and spores. The berries may look dry in the field but become soft and watery when stored after harvesting. Fruit are exposed to the pathogen from plant debris in the field or from leaf spots caused by the same pathogen (Troncoso-Rojas and Tiznado-Hernández, 2014). In conventional blueberry production in the U.S., the primary way to reduce *Alternaria* spp. infections is to apply different classes of fungicides starting from bloom through harvest (Neugebauer et al., 2024). Several site-specific fungicides including quinone outside inhibitors (QoIs), succinate dehydrogenase inhibitors (SDHIs), demethylation inhibitors (DMIs), phenylpyrroles, and anilinopyrimidines (APs) are utilized in Georgia and elsewhere for Alternaria fruit rot control (Sial et al., 2023; Neugebauer et al., 2024).

Researchers recommend these fungicides for use in blueberries because of their efficacy against *Alternaria* spp. However, because these fungicides are used widely in a variety of crops, there is considerable selection pressure that can lead to the development of resistance to these fungicides, and resistance development can be a common issue (Deising et al., 2008). Frequent use of relatively few specific fungicides results in a high selection pressure. The pathogens that cause blueberry fruit rots have already been found to be resistant to some fungicides in different parts of the U.S. In a recent publication (Wang et al., 2022), sensitivity profiles of *A. alternata* isolates from blueberry fields to quinone outside inhibitors (QoIs), boscalid, fluopyram, fludioxonil, cyprodinil, and polyoxin D in California were examined. Out of 143 isolates, all were considered resistant to boscalid and sensitive to fludioxonil and cyprodinil while 32, 69, and 42 isolates were sensitive, low resistant, and resistant to fluopyram, respectively. In addition, 60 of the 143 isolates were QoI resistant. Fungicide resistance in *Colletotrichum gloeosporioides*, the pathogen responsible for anthracnose rot, has already been documented in blueberry in Georgia (Ali et al., 2019), with resistance to pyraclostrobin, boscalid, and thiophanate-methyl identified. To maximize efficacy of the fungicide spray program and minimize further resistance development, it is important to know the current fungicide resistance status of the *Alternaria* spp. in blueberries and to monitor any early shifts in the pathogen’s sensitivity. An assessment of the resistance status against currently utilized fungicides may provide an opportunity to make changes to the fungicide recommendations to improve management of Alternaria rot. There is no data available on the sensitivity status of the *Alternaria* spp. on blueberries in Georgia. While EC_50_ values are usually the determinant of the sensitivity status of a particular fungicide against a specific pathogen, there are no EC_50_ values from Georgia available for any fungicides against the *Alternaria* spp. causing disease on blueberries. Therefore, in our study, *Alternaria* species associated with blueberry fruit rot in Georgia were isolated and identified with morphological and molecular methods, verified as pathogens via pathogenicity testing, and utilized in fungicide sensitivity assays to determine EC_50_ values for fludioxonil, fluazinam, metconazole, cyprodinil, pydiflumetofen, boscalid, and pyraclostrobin.

## 2 Materials and methods

### 2.1 Pathogen isolation

For isolation of *Alternaria* spp., blueberries were collected from multiple locations within major blueberry-producing counties in Georgia including Appling, Bacon, Brantley, and Pierce (**Supplementary Figure S1**). Fungal isolates were cultured from symptomatic (rotting) berries on acidified ¼ strength potato dextrose agar (AqPDA). Agar was acidified using 184 μl lactic acid (85% w/w) per liter. AqPDA plates were incubated for 2-4 days at room temperature (~23°C) to allow for fungal growth. Once fungal growth was observed, pure culture isolates were obtained by hyphal tip cutting and maintained on AqPDA for 7 additional days. To store isolates, mycelial plugs (4 mm) were cut from the leading edges of fungal colonies and placed in 20% glycerol at 4°C.

### 2.2 Morphological identification

For morphological identification, isolates were first cultured on potato dextrose agar (PDA) for 3-5 days. Mycelial plugs (4 mm) taken from the edge of each colony were transferred to two 9 cm plastic Petri dishes, one containing V8 agar and the other one containing PDA. Plates were sealed with parafilm and incubated in the dark at 25°C for 7 days. After this, Petri dishes with V8 agar were unsealed and kept at 25°C in 12hr-12hr light-darkness conditions for 2 to 3 additional days. Conidial characteristics were observed from the V8 agar plates under a light microscope at 400x magnification. Photographs of the plates were taken, and conidial lengths and widths were measured.

### 2.3 Molecular identification

DNA was extracted from each of the 46 isolates from 7-day-old PDA cultures. The mycelium was scraped off using a sterile loop and placed into a 2-mL microcentrifuge tube containing approximately twenty 2-mm zirconia/silica ceramic beads (Research Products International, Mount Prospect, IL). After grinding the sample by shaking for 30 seconds in a Biospec Mini Beadbeater-8 (BioSpec Products, Bartlesville, OK), DNA was extracted using a CTAB (cetyltrimethylammonium bromide) extraction method (Doyle and Doyle, 1987). The ITS1 and ITS4 primer set (**Table 1**) was used to amplify the internal transcribed spacer (ITS) region containing ITS1-5.8S-ITS2 of nuclear ribosomal DNA (rDNA) (White et al., 1990). For further identification of *Alternaria* spp., primer pair ATPDF1 and ATPDR1 (**Table 1**) was used to amplify the gene encoding the plasma membrane ATPase (Lawrence et al., 2013). For a subset of isolates, additional primer pairs (**Table 1**) were used to amplify sequences of the *Alternaria* major allergen (*Alt a1*), calmodulin (CAL), and the second largest subunit of RNA polymerase II (RPB2) (Hong et al., 2005; Lawrence et al., 2013). For PCR, a total reaction volume of 30 μl was used, and each reaction contained 15 μl 2X PCR Master Mix (Promega, Madison, WI), approximately 200 ng of DNA, and 10 mM of each primer (1 μl each). PCR was performed using a Bio-Rad S1000 Thermal Cycler (Bio-Rad Laboratories, Hercules, CA) according to the published reaction conditions for each primer set (references in **Table 1**). PCR products were visualized in a 1% agarose gel stained with GelRed Nucleic Acid Stain (Biotium, Fremont, CA) using a Bio-Rad Molecular Image Gel Doc XR+ with Image Lab Software (Bio-Rad Laboratories, Hercules, CA). Amplified PCR products were purified using the E.Z.N.A. Cycle Pure Kit (Omega Bio-tec, Inc., Norcross, GA) and Sanger sequenced in both directions by Eurofins Genomics (Louisville, KY). Isolates were initially confirmed as belonging to *Alternaria* spp. by comparison of obtained ITS sequences with publicly available *Alternaria* spp. sequences in the GenBank database using the BLASTn tool (http://www.ncbi.nlm.nih.gov/BLAST/).

**Table 1:** Primers used in this study.

Primer Name	Sequence (5’-3’)	Reference
ITS1	TCCGTAGGTGAA CCTGCGG	White et al., 1990
ITS4	TCCTCCGCTTA TTGATATGC	
ATPDF1	ATCGTCTCCATGACCGAGTTCG	Lawrence et al., 2013
ATPDR1	TCCGATGGAGTTCATGATAGCC	
Alt-for	ATGCAGTTCACCACCATCGC	Hong et al., 2005
Alt-rev	ACGAGGGTGAYGTAGGCGTC	
CALDF1	AGCAAGTCTCCGAGTTCAAGG	Lawrence et al., 2013
CALDR1	CTTCTGCATCATCAYCTGGACG	
RPB2DF	ACCGACACACAAATGCTGGAGC	Lawrence et al., 2013
RPB2DR	CAAGACCCCAATGAGAGTTGTG	
SdhBF6	AAGGAAGATCGCAAGAAGCTC	Avenot et al., 2008a
SdhBR6	AATGGCTAGCGCAGGGTTCA	
SdhC-(A-G) F1	CACCTGGCCATCTACAAGC	Avenot et al., 2009
SdhC-(A-G) R1	TGGTTCTTGAAACCAATACCG	
SdhD(C-A) S1	CCACTGGAGCTTCGAGAGGA	
SdhD(C-A) R1	GCTGTTCGAGTCTTGGGAAC	
cytb2f	CTATGGATCTTACAGAGCAC	Vega and Dewdney, 2014
DTRcytb2-INTr	GTATGTAACCGTCTCCGTC	

### 2.4 Phylogenetic analysis

For identification of isolates to the species level, a phylogenetic analysis was performed using the plasma membrane ATPase gene sequence for each of these isolates and 56 reference isolates (**Supplementary Table S1**) previously classified as belonging to 49 different *Alternaria* spp. (Lawrence et al., 2013; Woudenberg et al., 2015; Zhu and Xiao, 2015; Luo et al., 2018; Elfar et al., 2019; Qian et al., 2022; Elfar et al., 2023; Yan et al., 2024). were selected to allow for classification of isolates to the species level. ATPase sequences were initially aligned with the CLUSTAL X program (Thompson et al., 1997) and further edited in MEGA7 (Kumar et al., 2016). Evolutionary analyses were conducted in MEGA7. The evolutionary history was inferred by using the Maximum Likelihood method based on the Tamura-Nei model (Tamura and Nei, 1993). Initial tree(s) for the heuristic search were obtained automatically by applying Neighbor-Join and BioNJ algorithms to a matrix of pairwise distances estimated using the Maximum Composite Likelihood (MCL) approach and then selecting the topology with the superior log likelihood value. A discrete Gamma distribution was used to model evolutionary rate differences among sites (5 categories (+*G*, parameter = 0.3447)). All positions with less than 95% site coverage were eliminated. That is, fewer than 5% alignment gaps, missing data, and ambiguous bases were allowed at any position.

### 2.5 Pathogenicity testing

In total, 22 isolates were selected representing all the locations and isolated species of *Alternaria* for pathogenicity confirmation based on Zhu and Xiao (2015) with slight modifications. Store-bought, firm, ripe organic blueberries were selected for inoculation experiments. Berries were surface sterilized by briefly dipping twice in 70% ethanol, once in 0.5% sodium hypochlorite, and twice in sterile distilled water. Air dried berries were fixed to the bottom of clamshells with double-sided tape with the stem-scar facing up. Spore suspensions prepared as described in section 2.2 were prepared and standardized to a concentration of 10^5^ spores per milliliter of water after counting the number of spores with a hemocytometer. Each berry was inoculated with 20 µl of spore suspension on the stem scar site. For each isolate, three clamshells which each contained 9 berries (27 berries total) were inoculated. The clamshells were placed in a sealed plastic box at room temperature, and two sterile paper towels soaked with sterile distilled water were placed at the bottom of each box to ensure humid conditions. After 7 days, the berries were visually rated for disease incidence (as the presence of any spores or mycelium) and severity on a scale of 0 to 5 based on Saito et al. (2016) with a slight modification (**Supplementary Figure 2**). The Disease Index (DI) was calculated according to Fu et al. (2020). The DI was computed using the formula:


$$DI=\frac{\sum \left(n\times corresponding\;DS\right)\;}{N\times 5}\times 100$$

where, DI = Disease Index, DS = Disease Severity, n = the number of berries corresponding to each disease rating, N = the total number of berries inoculated. Re-isolation from diseased berries and identification of the obtained isolates was carried out to fulfill Koch’s postulates.

### 2.6 Fungicide sensitivity assessment

For sensitivity testing, seven technical grade fungicides were used including cyprodinil (purity 99.9%), fludioxonil (99.5%), fluazinam (98.4%), metconazole (98.9%), pydiflumetofen (99.2%), boscalid (97.1%), and pyraclostrobin (98.5%) from Sigma-Aldrich Corp. (St. Louis, MO, USA) and dissolved in acetone for the preparation of stock solutions (1,000 µg/mL). PDA was amended with each fungicide to final concentrations ranging from 0.01 to 100 µg/mL (**Table 2**) alongside non-amended control plates. These concentrations were used to ensure a fungal growth inhibition range from only slightly to almost complete inhibition. Fungicide sensitivity tests were repeated two times for each of the 46 isolates and each test consisted of two Petri plates with each concentration of each fungicide. Mycelial growth inhibition assays were carried out for each of the fungicides, except pyraclostrobin, for the determination of the EC_50_ values (50% mycelial growth inhibition).

**Table 2:** Fungicidal product commonly utilized for Alternaria fruit rot and leaf spot control in blueberry production, active ingredient, FRAC mode of action, and concentrations of active ingredient used in the mycelial growth inhibition assays conducted as part of this study.

Trade Name	Active Ingredient	Group (FRAC MoA)	Concentration Used (μg/μl)
Quash	Metconazole	DMI (FRAC 3)	0.01, 0.05, 0.1, 0.5, 1.0
Omega 500F	Fluazinam	2,6-dinitroanilines (FRAC 29)	0.001, 0.003, 0.01, 0.03, 0.1, 0.3
Pristine	Pyraclostrobin	QoI (FRAC 11)	0.01, 0.1, 1.0, 10
	Boscalid	SDHI (FRAC 7)	0.5, 1.0, 5.0, 10, 50, 100
Switch 62.5WG	Cyprodinil	Anilopyrimidines (FRAC 9)	0.1, 0.5, 1.0, 5.0, 10
	Fludioxonil	Phenylpyrroles (FRAC 12)	0.01, 0.05, 0.1, 0.5, 1.0, 5.0
Miravis Prime	Pydiflumetofen	SDHI (FRAC 7)	0.005, 0.01, 0.05, 0.1, 0.5, 1, 5
	Fludioxonil	Phenylpyrroles (FRAC 12)	0.01, 0.05, 0.1, 0.5, 1.0, 5.0

For pyraclostrobin, spore germination inhibition assays were performed. For mycelial growth inhibition assays, mycelial plugs (4 mm in diameter) were removed from the margins of colonies grown on PDA and placed upside-down on the fungicide-amended and fungicide-free PDA media which were incubated at 25 ± 1°C. After 4-5 days, the colony growth of each isolate was measured (the 4 mm diameter of the inoculation plug was subtracted from the colony diameter) and the percent inhibition (PI) values for each fungicide rate was calculated using the formula:


$$PI=\;\frac{a-b}{a}\times 100$$

where a = colony growth of the control plate, and b = colony growth of the fungicide-amended plate.

The EC_50_ for each isolate was determined based on the percent inhibition on each of the different fungicide concentrations used. Relative growth inhibition was regressed against the log_10_ fungicide concentration using Statistical Analysis System (SAS Institute Inc., Cary, NC) for calculation of the EC_50_ values. For the pyraclostrobin spore germination inhibition assay, spores from each isolate of *Alternaria* spp. were produced, scraped off with sterile plastic loops, suspended in 10% tween 20, and adjusted to 10^5^ spores per milliliter using a hematocytometer. Water agar plates were prepared for the four tested concentrations (0.01, 0.1, 1, and 10 μg/μL) of pyraclostrobin. In the control plate, no pyraclostrobin was added. Then, 100 μL of the spore suspension was added and dispersed onto each of these plates. After incubation of these plates at 28°C for 24 hours, germination of 100 randomly selected spores from each plate was observed, counting those germinated and those not germinated. Based on these observations, percent inhibition (PI) values for each of the fungicide rates were calculated using the previous formula where a = number of spores germinated in the control plate, and b = number of spores germinated in the fungicide-amended plate. The EC_50_ values were calculated in the same way as for the mycelial growth inhibition assay using Statistical Analysis System (SAS Institute Inc., Cary, NC).

To examine correlations between EC_50_ values for the two SDHI fungicides examined (boscalid and pydiflumetofen), the Pearson correlation coefficient (r) and the associated p-value were computed using SigmaPlot 16 (Systat Software Inc., San Jose, CA). For pydiflumetofen, since baseline information establishing the thresholds for resistance and reduced sensitivity were not available, the frequency distribution of the EC_50_ values were further subjected to a Shapiro-Wilk test for normality (JMP^®^, Version 17.2.0. SAS Institute Inc., Cary, NC, 1989–2023) to evaluate for the presence of values that may indicate reduced sensitivity to this fungicide.

### 2.7 Mutation identification in fungicide-resistant *Alternaria* spp. isolates

To determine if the fungicide-resistant *Alternaria* spp. isolates possess mutations known to be associated with fungicide resistance, sequencing the fungal *sdhB*, *sdhC*, *sdhD*, and *cytB* genes was carried out via PCR with specific primers (**Table 1**). A total reaction volume of 20 μl was used, and each reaction contained 10 μl 2X PCR Master Mix (Promega, Madison, WI), approximately 200 ng of genomic DNA, and 10 mM of each primer (1 μl each). PCR was performed using a Bio-Rad S1000 Thermal Cycler (Bio-Rad Laboratories, Hercules, CA) according to the previously published reaction conditions for each primer set (Avenot et al., 2008a, 2009; Vega and Dewdney, 2014). Amplified PCR products were purified using the E.Z.N.A. Cycle Pure Kit (Omega Bio-tec, Inc., Norcross, GA) and Sanger sequenced in both directions by Eurofins Genomics (Louisville, KY).

## 3 Results

### 3.1 Morphological characteristics

**Figure 1:**
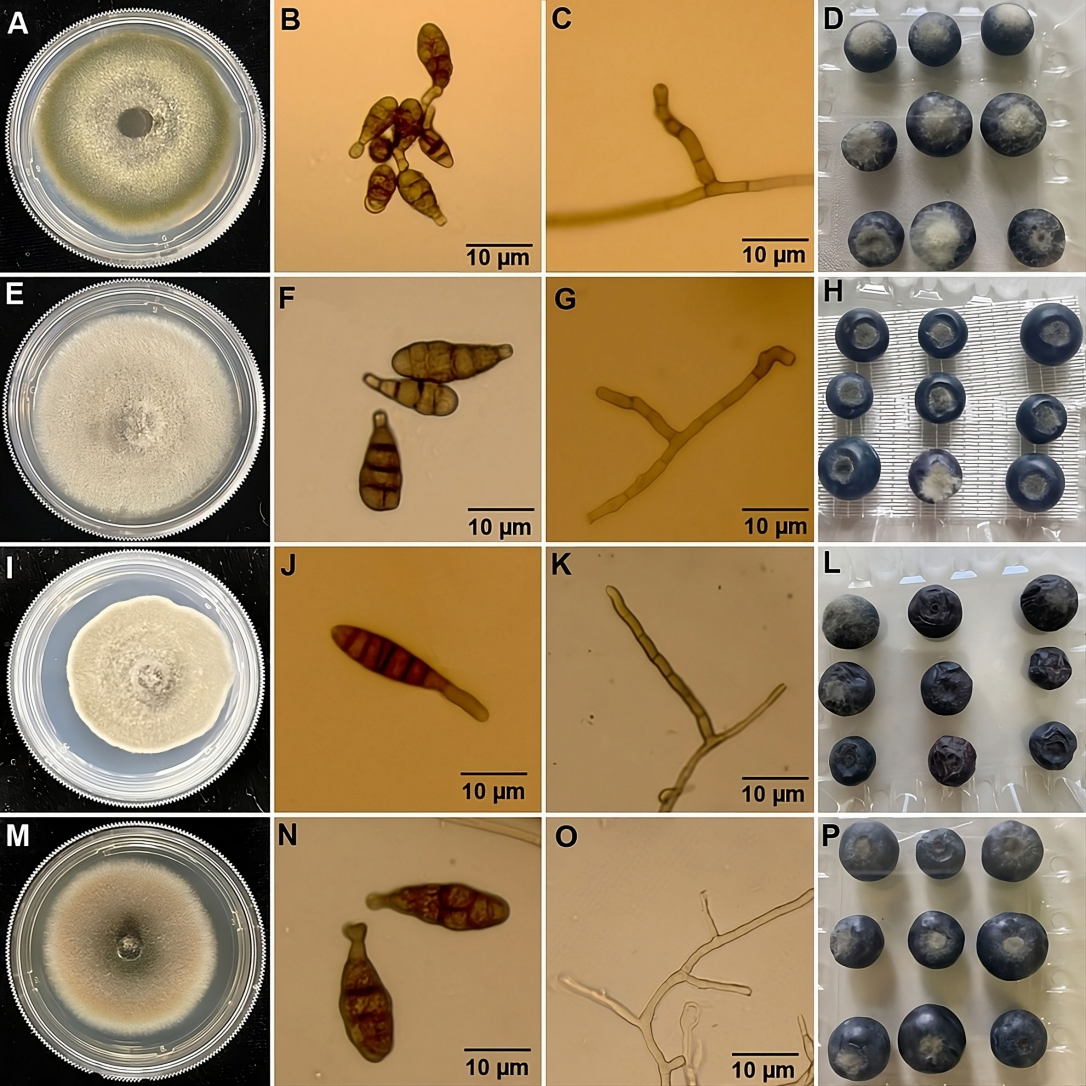
Morphological features & pathogenicity testing of representative isolates from blueberry of each *Alternaria* species. **(A)** Colony morphology on potato dextrose agar (PDA) after 5 days incubation at 22°C; **(B)** conidia; **(C)** conidiophore; **(D)** symptoms on blueberry fruits following inoculation with representative isolates after 7 days of incubation at 22°C. Based on morphological characteristics and phylogenetic analysis, the isolate depicted in panels **(A–D)** was identified as *A. alternata* (MB21-397); **(E–H)** were identified as *A. tenuissima* (MB21-456); **(I–L)** were identified as *A. dumosa* (MB21-363); and **(M–P)** were identified as *A. limoniasperae* (MB21-475).

A total of 46 *Alternaria* spp. were isolated from rotting berries from 16 commercial blueberry farms in southeastern Georgia (**Supplementary Table S2**). Growth characteristics and conidial morphology of these isolates were consistent with those of *A. alternata, A. tenuissima*, *A. dumosa* and *A. limoniasperae* as described by Simmons (1967; 2007). Among these, 43 of 46 isolates were identified as *A. alternata*. These isolates were initially greyish green to olive brown in color on the PDA plates (45–47 mm in 5 days) (MB21-397; **Figure 1A**) and whitish green on V8 agar (35–40 mm in 5 days) (data not shown). The conidia were generally ovoid to ellipsoid and ranged from 8.7–21.2 × 7.2–11.3 µm in size (n=30) with one to four transverse and zero to two longitudinal septa per conidium (**Figure 1B**). The conidiophores of these isolates were singular, short, and measured 17.9–60.5 × 2.8–6.6 µm in size (n=15) (**Figure 1C**). One isolate (MB21-456) was identified as *A. tenuissima* and was characterized by grayish color on PDA (50–52 mm in 5 days) (**Figure 1E**) and whitish gray on V8 (45–50 mm in 5 days) (data not shown). The conidia were ovoid with a tapering apical beak and a size of 11.5–31.5 × 5.1–12.7 µm (n=30), with one to five transverse and zero to one longitudinal septa per conidium (**Figure 1F**). Conidiophores 15.6–57.4 × 3.1–6.8 µm (n=15) were arising singly and short (**Figure 1G**). Another isolate (MB21-363) was identified as *A. dumosa* and was characterized by brown color on PDA (35–40 mm in 5 days) (**Figure 1I**) and whitish cottony gray on V8 (43–47 mm in 5 days) (data not shown). Conidia were ovoid size of 17.5–41.5 × 4.5–8.4 µm (N=30), with one to seven transverse and zero to one longitudinal septa per conidium (**Figure 1J**). The conidiophores of this isolate were singular, long, and measured 37.5–115.3 × 3.0–4.3 µm (n=15) (**Figure 1K**). The final isolate (MB21-475) was identified as *A. limoniasperae* and was light brown-green on PDA (43-45 mm in 5 days) (**Figure 1M**) and whitish grey on V8 (45–50 mm in 5 days) (data not shown). Conidia were narrow-ellipsoid to ovoid 25.3–45.3× 6.5–8.9 µm (n=30) with one to five transverse and one to two longitudinal septa per conidium (**Figure 1N**). The primary conidiophores were large 65–110 × 3–5 μm (n=10), but the secondary conidiophores were short 3–21×2–4 µm (n=10) (**Figure 1O**).

### 3.2 Molecular identification and phylogenetic analysis

**Figure 2:**
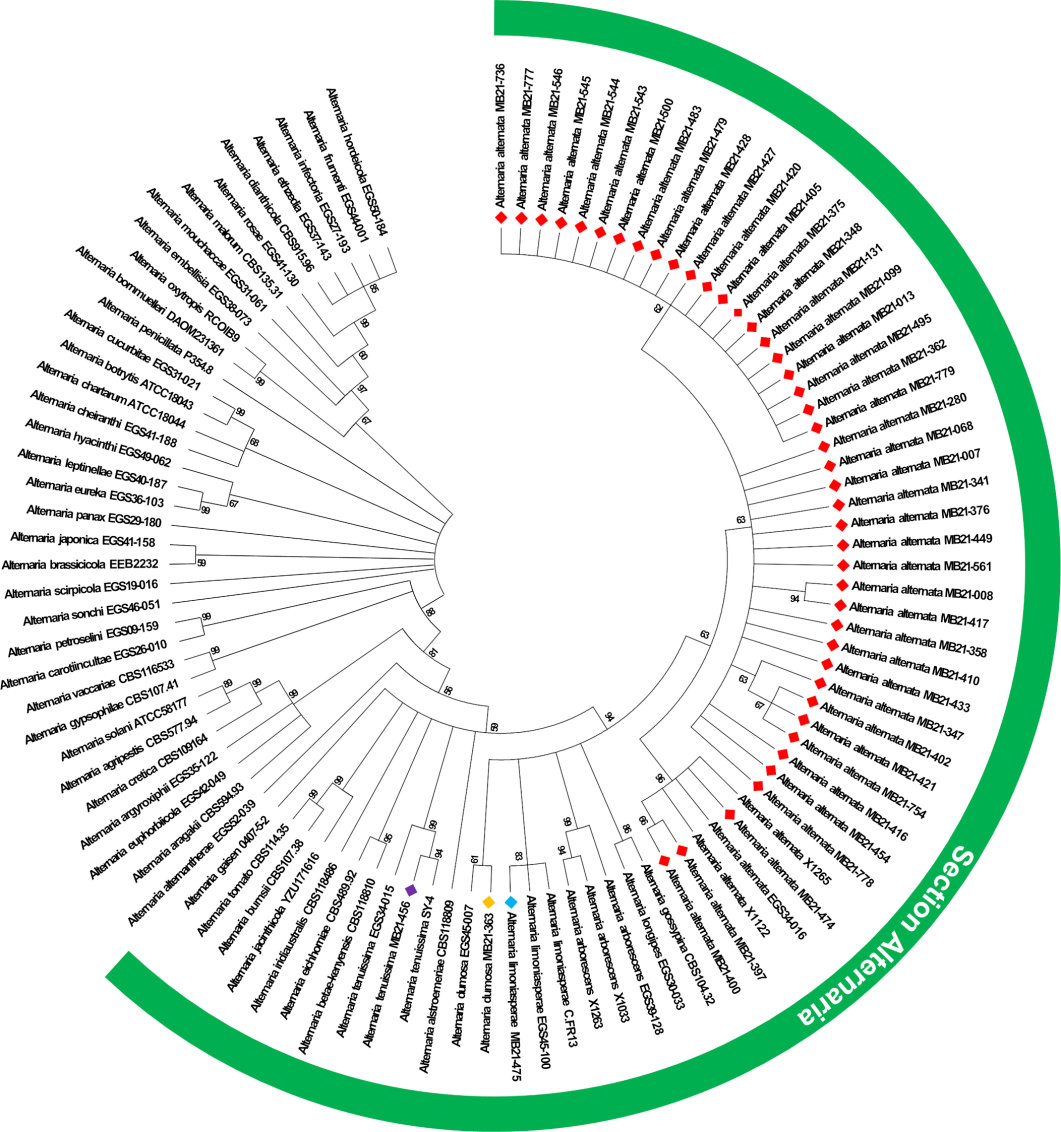
Maximum likelihood phylogenetic tree based on ATPase gene sequences of isolates from this study alongside other isolates from the genus *Alternaria*. Isolates from this study are indicated by a colored diamond. A red diamond indicates isolates identified as *A. alternata*, and the light blue, orange, and violet diamonds indicate isolates of *A. limoniasperae*, *A dumosa*, and *A. tenuissima*, respectively. Isolates from species within the section *Alternaria* (which is within the genus *Alternaria*) are indicated by the green semi-circle.

The results of ITS sequencing (Genbank accession numbers OR041698-OR041743) confirmed all 46 isolates as belonging to *Alternaria* species. Sequences obtained from other gene regions further confirmed this assessment (Genbank accession numbers OR091105-OR091150 [ATPase], PP662487-PP662508 [*Alt a1*], PP662470-PP662475 [CAL], and PP662476-PP662481 [RPB2]). Phylogenetic analysis of *Alternaria* spp. isolates using the ATPase gene enabled the identification of all isolates to the species level (**Figure 2**). Based upon this analysis, all isolates from this study segregated with isolates from section *Alternaria* within the genus *Alternaria*, with the vast majority of isolates from this study forming a single clade with reference isolates of *A. alternata*. In total, 43 isolates from this study were identified as *A. alternata* and the remaining isolates were classified as *A. tenuissima* (n=1), *A. dumosa* (n=1), and *A. limoniasperae* (n=1) (**Figure 2**).

### 3.3 Pathogenicity testing

Inoculation of detached blueberry fruit with *Alternaria* isolates yielded lesions of dark brown mycelium growth and rotten berries during the seven days following initial inoculation (**Figures 1D, H, L, P**). To fulfill Koch’s postulates, re-isolation from diseased berries and identification of the obtained isolates was performed to confirm the presence of *Alternaria* spp. in the rotting berries. Isolates of *A. alternata* and *A. tenuissima* had higher disease indexes and equal or higher disease incidences on blueberry fruits as compared to the isolates of *A. dumosa* and *A. limoniasperae*. The disease indexes and disease incidences (%) ranged from 40.0–82.2 and 88.9-100%, respectively, for the 19 A*. alternata* isolates tested, and were 54.1 and 100%, 19.3 and 85.2%, and 39.3 and 88.9% for the isolates of *A. tenuissima*, *A. dumosa*, and *A. limoniasperae*, respectively (**Table 3**).

**Table 3:** Pathogenicity test results including disease severity index and incidence (%) of selected *Alternaria* spp. isolates.

Species Identity	Isolate Name	Disease incidence (%)	Disease Severity Index
*A. alternata*	MB21-013	92.6	45.9
	MB21-068	100	57.8
	MB21-099	100	79.3
	MB21-348	100	48.9
	MB21-362	100	70.4
	MB21-397*	100	65.2
	MB21-402	96.3	58.5
	MB21-410	100	66.7
	MB21-417	100	66.7
	MB21-421	88.9	46.7
	MB21-449	100	68.9
	MB21-454	100	58.5
	MB21-495	96.3	54.8
	MB21-500	100	48.9
	MB21-543	88.9	40.0
	MB21-546	100	82.2
	MB21-561	96.3	51.1
	MB21-736	96.3	68.1
	MB21-777	100	55.6
*A. tenuissima*	MB21-456*	100	54.1
*A. dumosa*	MB21-363*	85.2	19.3
*A. limoniasperae*	MB21-475*	88.9	39.3

*isolates depicted in **Figure 2**

### 3.4 Fungicide sensitivity of *Alternaria* spp. isolates

**Figure 3:**
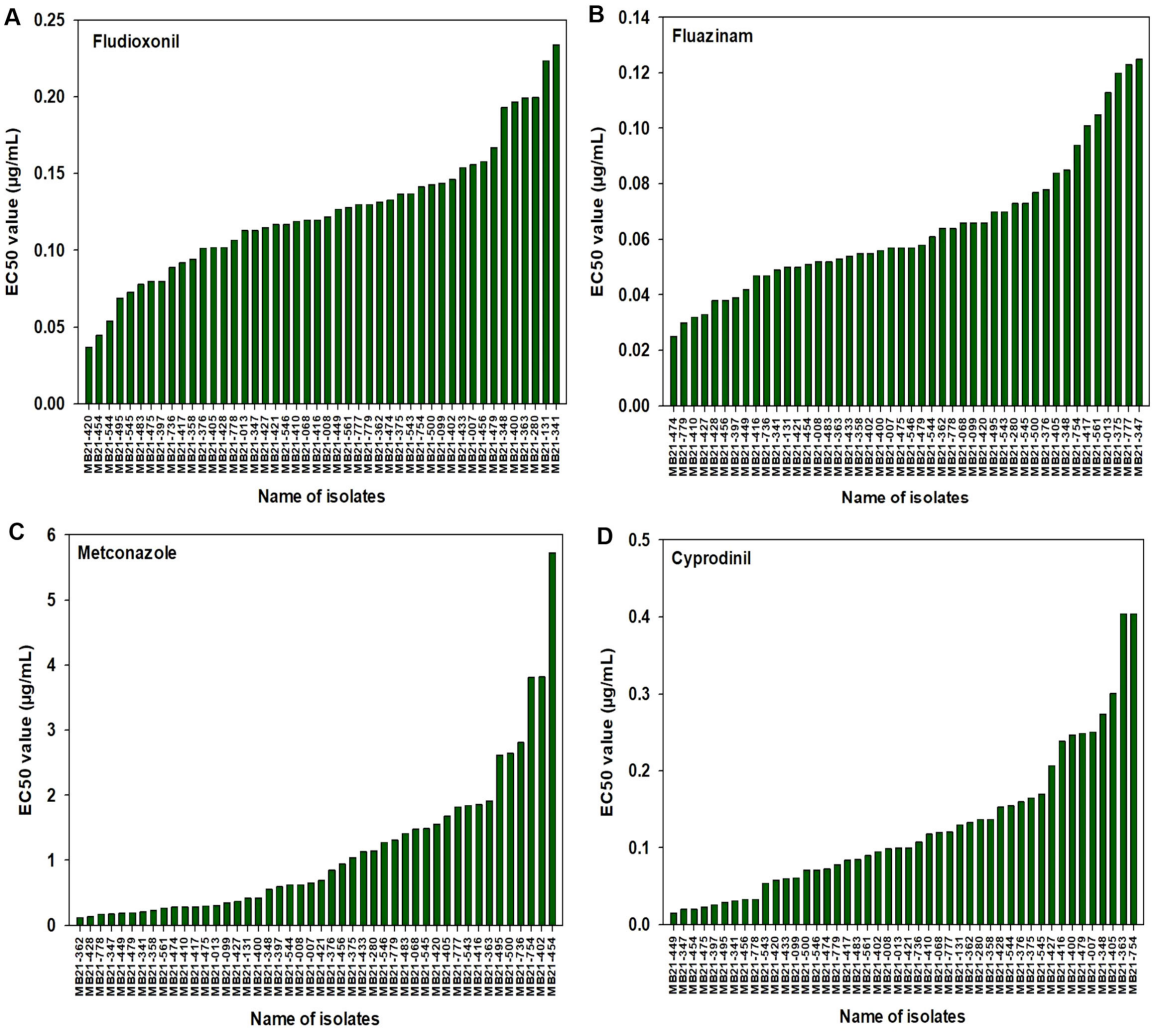
EC_50_ values determined based on a mycelial growth inhibition assay from all 46 isolates used in this study for: **(A)** fludioxonil, **(B)** fluazinam, **(C)** metconazole, and **(D)** cyprodinil. Results are depicted for 43 *A. alternata*, one *A. tenuissima* (MB21-456), one *A. dumosa* (MB21-363), and one *A. limoniasperae* (MB21-475).

**Figure 4:**
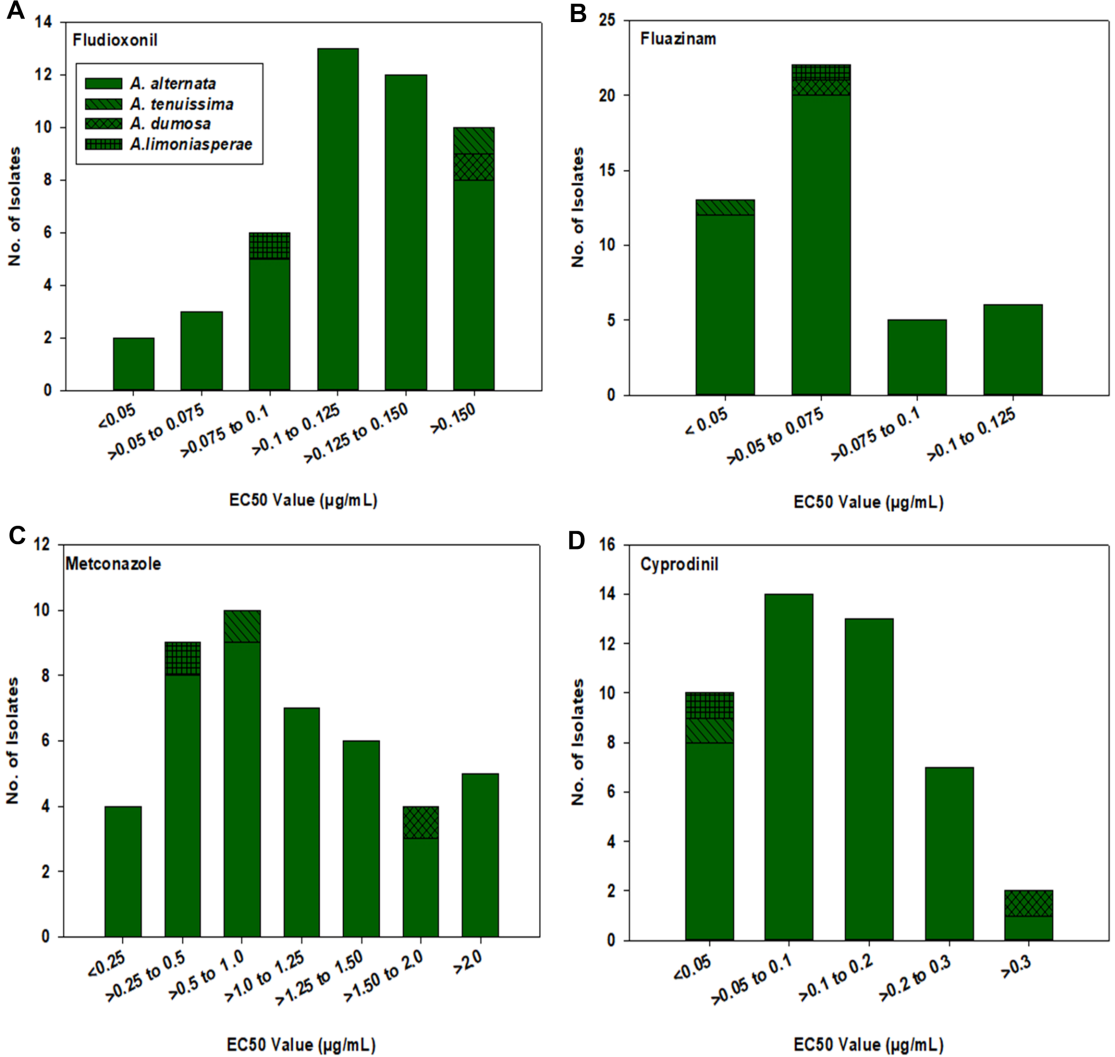
Frequency distribution of EC_50_ values (based on a mycelial growth inhibition assay) for all 46 *Alternaria* spp. isolates from this study for: **(A)** fludioxonil, **(B)** fluazinam, **(C)** metconazole, and **(D)** cyprodinil. Results are depicted for 43 *A. alternata*, one *A. tenuissima* (MB21-456), one *A. dumosa* (MB21-363), and one *A. limoniasperae* (MB21-475) as indicated by the figure legend.

For fludioxonil, the EC_50_ values for the 43 A*. alternata* isolates ranged from 0.037 to 0.234 µg/mL (**Figure 3A**). The mean EC_50_ value for these isolates was 0.124 µg/mL with a standard deviation of 0.043 µg/mL. The EC_50_ values for the other three isolates were 0.199 µg/mL for MB21-363 (*A. dumosa*), 0.158 µg/mL for MB21-456 (*A. tenuissima*), and 0.080 µg/mL for MB21-475 (*A. limoniasperae*). As a whole, the fludioxonil EC_50_ values showed a near-unimodal distribution pattern skewed toward the left (low values) with a few isolates being less sensitive than most other isolates (**Figure 4A**).

For fluazinam, the EC_50_ values for the 43 A*. alternata* isolates ranged from 0.025 to 0.125 µg/mL (**Figure 3B**). The mean EC_50_ value for this fungicide was 0.065 µg/mL with a standard deviation of 0.025 µg/mL. The EC_50_ values for the other three isolates were 0.053 µg/mL for MB21-363 (*A. dumosa*), 0.038 µg/mL for MB21-456 (*A. tenuissima*), and 0.057 µg/mL for MB21-475 (*A. limoniasperae*). The frequency distribution for this fungicide showed a near-unimodal pattern where most isolates had EC_50_ values less than 0.01 µg/mL (**Figure 4B**).

For metconazole, the EC_50_ values of the 43 A*. alternata* isolates ranged from 0.125 to 5.729 µg/mL (**Figure 3C**). The mean EC_50_ value of these isolates for this fungicide was 1.153 µg/mL with a standard deviation of 1.194 µg/mL. The EC_50_ values for the other three isolates were 1.914 µg/mL for MB21-363 (*A. dumosa*), 0.945 µg/mL for MB21-456 (*A. tenuissima*), and 0.300 µg/mL for MB21-475 (*A. limoniasperae*). For this fungicide, the frequency distribution of EC_50_ values had a near-unimodal pattern (**Figure 4C**).

For cyprodinil, the EC_50_ values of the 43 A. *alternata* isolates ranged from 0.015 to 0.404 µg/mL (**Figure 3D**). The mean EC_50_ value was 0.124 µg/mL with a standard deviation of 0.086 µg/mL. EC_50_ values for the other three were 0.404 µg/mL for MB21-363 (*A. dumosa*), 0.033 µg/mL for MB21-456 (*A. tenuissima*), and 0.023 µg/mL for MB21-475 (*A. limoniasperae*). The frequency distribution for this fungicide was unimodal and skewed slightly towards the higher values having a small number of isolates with higher EC_50_ values (**Figure 4D**).

**Figure 5:**
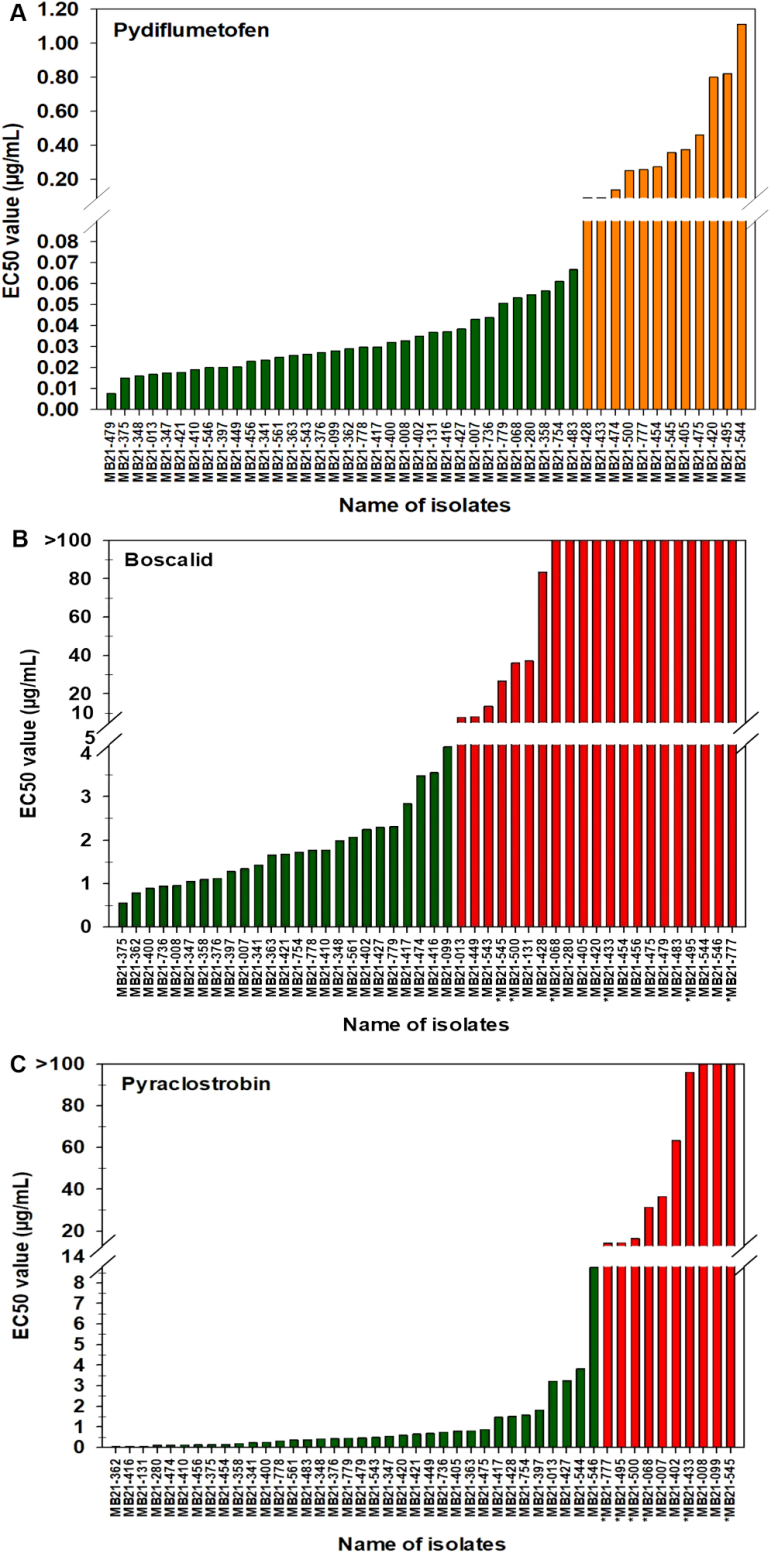
EC_50_ values from all 46 isolates from this study determined for: **(A)** pydiflumetofen, **(B)** boscalid, and **(C)** pyraclostrobin. Values were determined based on a mycelial growth inhibition assay for pydiflumetofen and boscalid and via a spore germination for pyraclostrobin. Results are depicted for 43 *A. alternata*, one *A. tenuissima* (MB21-456), one *A. dumosa* (MB21-363), and one *A. limoniasperae* (MB21-475). An asterisk (*) indicates those isolates that were double resistant to boscalid and pyraclostrobin. Green color indicates sensitive isolates, orange color indicates isolates with reduced sensitivity, and red color indicates resistant isolates.

**Figure 6:**
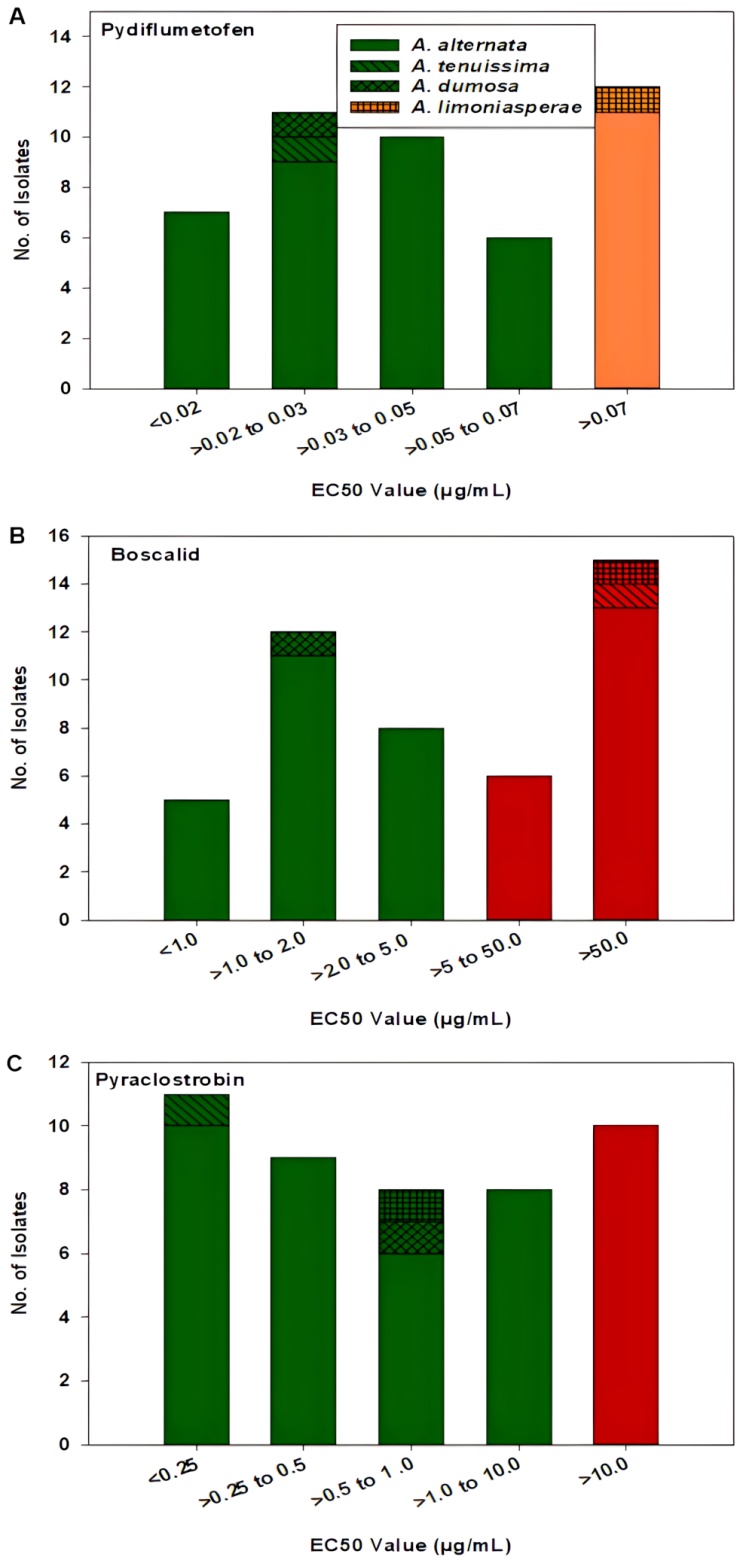
Frequency distribution of EC_50_ values for all 46 *Alternaria* spp. isolates from this study for **(A)** pydiflumetofen, **(B)** boscalid, and **(C)** pyraclostrobin. Values were determined based on a mycelial growth inhibition assay for pydiflumetofen and boscalid and via a spore germination for pyraclostrobin. Results are depicted for 43 *A. alternata*, one *A. tenuissima* (MB21-456), one *A. dumosa* (MB21-363), and one *A. limoniasperae* (MB21-475) as indicated by the figure legend. Green color indicates sensitive isolates, orange color indicates isolates with reduced sensitivity, and red color indicates resistant isolates.

For pydiflumetofen, the 43 A*. alternata* isolates had EC_50_ values ranging from 0.008 to 1.114 µg/mL (**Figure 5A**). The mean value was 0.131 µg/mL with a standard deviation of 0.238 µg/mL. The EC_50_ values for the other three isolates were 0.026 µg/mL for MB21-363 (*A. dumosa*), 0.023 µg/mL for MB21-456 (*A. tenuissima*), and 0.463 µg/mL for MB21-475 (*A. limoniasperae*). The frequency distribution of the EC_50_ values for this fungicide did not fit a normal distribution. Since only data ranging from 0.008 to 0.067 µg/mL passes the Shapiro-Wilk goodness of fit test where the Shapiro–Wilk test statistic (W) is 0.93 (α = 0.05), the EC_50_ values higher than 0.067 were considered to have reduced sensitivity to pydiflumetofen. Based on this parameter, there were 12 isolates (11 A*. alternata* and 1 A*. limoniasperae*) with reduced sensitivity to pydiflumetofen (**Figure 6A**).

For boscalid, EC_50_ values were found to vary greatly among the 43 A*. alternata* isolates, ranging from 0.551 to greater than 100 µg/mL. On the basis that EC_50_ values above 5 µg/mL represent resistant isolates (Wang et al., 2022), there were 19 resistant and 24 sensitive isolates among the 43 collected *A. alternata* (**Figure 5B**). The EC_50_ values of the sensitive isolates ranged from 0.551 to 4.157 µg/mL, with a mean of 1.805 µg/mL and standard deviation of 0.941 µg/mL. For the resistant isolates, EC_50_ values ranged from 7.861 to greater than 100 µg/mL. Among these, there were 12 isolates that were extremely resistant, having EC_50_ values greater than 100 µg/mL. Isolate MB21-363 (*A. dumosa*) was sensitive to boscalid, with an EC_50_ value of 1.657 µg/mL. By contrast, isolates MB21-456 (*A. tenuissima*) and MB21-475 (*A. limoniasperae*) were resistant to boscalid with EC_50_ values of greater than 100 µg/mL. The frequency distribution of the EC_50_ values for boscalid showed a clear bimodal pattern with a shift towards higher EC_50_ values (**Figure 6B**).

The EC_50_ values for pyraclostrobin were found to vary considerably among the 43 A*. alternata* isolates (**Figure 5C**) ranging from 0.040 to greater than 100 µg/mL. Assuming that EC_50_ values above 10 µg/mL represent resistance (Avenot and Michailides, 2015), there were ten isolates determined to be resistant and 33 isolates determined to be sensitive. Among the sensitive isolates, the EC_50_ values ranged from 0.040 to 8.762 µg/mL, with an average of 1.035 µg/mL and standard deviation of 1.691 µg/mL. By contrast, for the ten resistant isolates, the EC_50_ values ranged from 14.35 to greater than 100 µg/mL. Among these, three isolates were extremely resistant, with EC_50_ values greater than 100 µg/mL. Isolates MB21-363 (*A. dumosa*), MB21-456 (*A. tenuissima*), and MB21-475 (*A. limoniasperae*) were sensitive to pyraclostrobin with EC_50_ values of 0.81 µg/mL, 0.13 µg/mL, and 0.88 µg/mL, respectively. For the 46 *Alternaria* spp. isolates, the frequency distribution of the EC_50_ values for pyraclostrobin showed a bimodal pattern suggesting a shift towards higher EC_50_ values (**Figure 6C**). Of the ten *Alternaria* isolates found to be resistant to pyraclostrobin, six isolates (MB21-068, MB21-433, MB21-495, MB21-500, MB21-545, MB21-777) from three locations (sites 5 and 7 in Bacon County, and site 14 in Pierce County), were also resistant to boscalid (**Table 4**). Furthermore, five of these six isolates (all isolates except MB21-068) also demonstrated reduced sensitivity to pydiflumetofen (**Figures 5**, **6**).

**Table 4:** Frequency of boscalid and/or pyraclostrobin sensitivities of *Alternaria* spp. isolates collected from blueberry sites in Georgia in this study.

County	Site Number	No. of Isolates	Boscalid Sensitivity (%)		Pyraclostrobin Sensitivity (%)		Resistance to both
			Sensitive	Resistant	Sensitive	Resistant	
Appling	1	3	3	0	2	1	0
	2	2	2	0	2	0	0
Bacon	3	5	5	0	5	0	0
	4	2	2	0	2	0	0
	5	14	3	11	12	2	2
	6	1	1	0	1	0	0
	7	4	2	2	3	1	1
	8	1	1	0	1	0	0
Brantley	9	1	0	1	1	0	0
	10	1	1	0	0	1	0
	11	2	2	0	0	2	0
Pierce	12	1	0	1	1	0	0
	13	2	2	0	2	0	0
	14	5	0	5	2	3	3
	15	1	1	0	1	0	0
	16	1	0	1	1	0	0
Totals	16	46	25 (54.3%)	21 (45.7%)	36 (78.3%)	10 (21.7%)	6 (13.0%)

### 3.5 Mutation identification in fungicide resistant isolates

#### 3.5.1 Mutations within *sdhB*, *sdhC*, and *sdhD* in isolates resistant to SDHI fungicides

**Figure 7:**
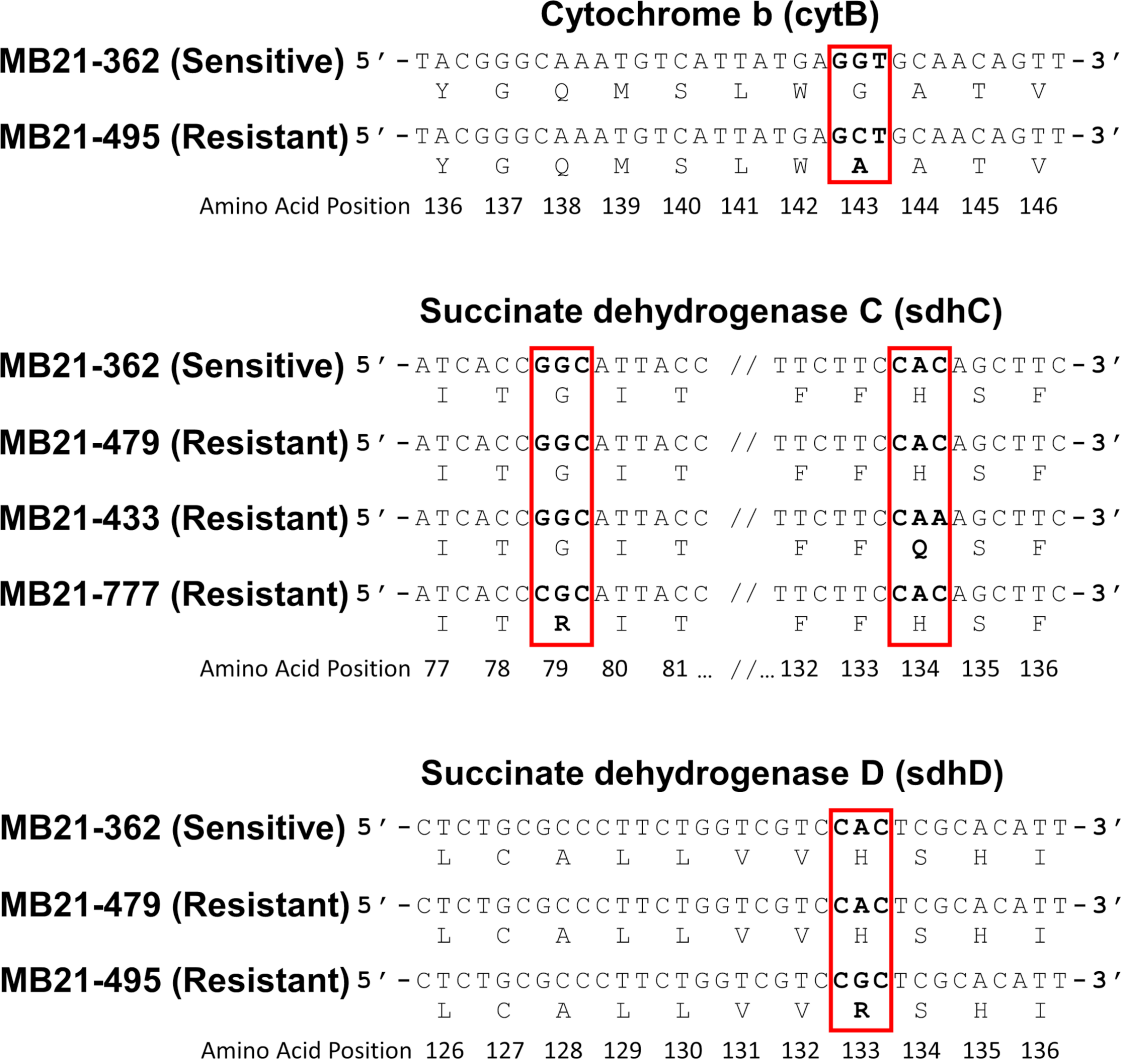
Partial nucleotide sequences of *cytB*, *sdhC*, and *sdhD* from selected *Alternaria* spp. isolates sequenced in this study alongside the corresponding amino acid within the predicted proteins. The sequences from pyraclostrobin-resistant isolates (including MB21-495; top panel) included a guanine (G) to cytosine (C) change at nucleotide position 123 within the sequenced portion of *cytB* which corresponds with an G143A amino acid change within the predicted protein sequence. Some boscalid-resistant isolates (including isolate MB21-433; middle panel), included a cytosine (C) to adenine (A) change at nucleotide position 228 within the sequenced portion of *sdhC* which corresponds with an H134Q amino acid change within the predicted protein sequence, while other boscalid-resistant isolates (including MB21-477; middle panel) included a guanine (G) to cytosine (C) change at nucleotide position 61 which corresponds to a G79R amino acid change. Within the sequenced portion of *sdhD*, additional boscalid-resistant isolates (including MB21-495; bottom panel) included an adenine (A) to guanine (G) change at nucleotide position 120 which corresponds to an H133R amino acid change with the predicted protein sequence. No other nucleotide differences expected to result in amino acid changes within cytB, sdhB, sdhC, or sdhD were noted between resistant and susceptible isolates.

Portions of *sdhB*, *sdhC*, and *sdhD* were sequenced from 16 selected *Alternaria* spp. isolates, including 12 boscalid-resistant isolates from four locations and four boscalid-sensitive isolates from three locations. Obtained sequences (Genbank accession numbers OR091065-OR091072 and PP620128-PP620135 [*sdhB*], OR091073-OR091080 and PP620136-PP620143 [*sdhC*], OR091081-OR091091 and PP620144-PP620148 [*sdhD*]; **Supplementary Table S2**) did not indicate any nucleotide changes within the sensitive isolates that would result in amino acid changes versus the previously-published *sdhB* (EU178851), *sdhC* (FJ437067), or *sdhD* (FJ437068) sequences of isolate AaY16, a known SDHI-sensitive *A. alternata* isolate (Avenot et al., 2008a, 2009). However, sequences from 9 of 12 boscalid-resistant isolates indicated nucleotide changes that would result in amino acid changes. Among these, all five boscalid-resistant isolates from site 14 (isolates MB21-495, MB21-500, MB21-543, MB21-544, and MB21-545) were found to possess a guanine at nucleotide position 120 within the obtained sequence of *sdhD*, which would result in an amino acid change at amino acid position 133 from histidine to arginine (H133R) (**Figure 7**; **Supplementary Table S2**). Three of five boscalid resistant isolates from site 5 (MB21-068, MB21-405, MB21-433) were found to possess an adenine at nucleotide position 228 within the obtained sequence of *sdhC*, which would result in an amino acid change at amino acid position 134 from histidine to glutamine (H134Q) (**Figure 7**; **Supplementary Table S1**). In addition, the sequence of *sdhC* from boscalid-resistant isolate MB21-777 from site 7 had a cytosine at nucleotide 61 resulting in an amino acid change at position 79 from glycine to arginine (G79R) (**Figure 7**; **Supplementary Table S1**). Mutations in either *sdhC* or *sdhD* were noted in all six isolates previously determined to be double-resistant to both boscalid and pyraclostrobin, with the H133R mutation found in isolates MB21-495, MB21-500, and MB21-545 (from site 14), the H134Q mutation found in isolates MB21-068 and MB21-433 (from site 5), and the G79R mutation found in isolate MB21-777 (from site 7).

#### 3.5.2 Mutation in *cytB* in pyraclostrobin resistant isolates

All ten pyraclostrobin-resistant isolates had a cytosine at nucleotide position 123 of the sequenced product (Genbank accession numbers OR091092-OR091104; **Supplementary Table S2**), whereas three sensitive isolates had a guanine at this position. This mutation results in a change from glycine to alanine (G143A) at amino acid position 143 (**Figure 7**).

### 3.6 Relationships between boscalid and pydiflumetofen isolate fungicide-sensitivities

**Figure 8:**
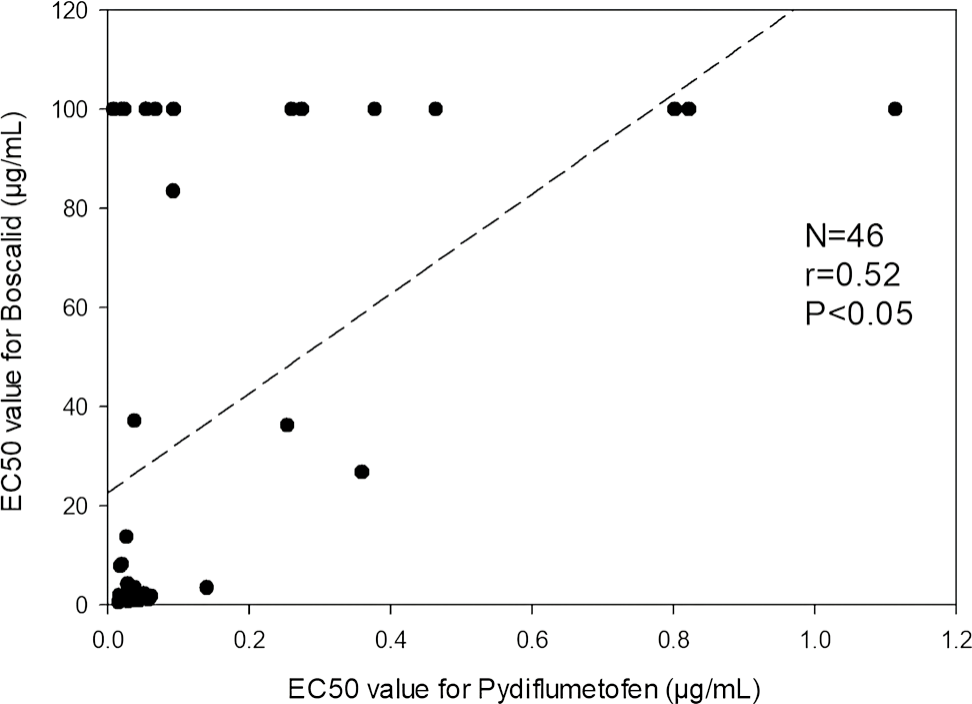
Graph depicting EC_50_ values for boscalid (y-axis) versus EC_50_ values for pydiflumetofen (x-axis) for each of the 46 *Alternaria* spp. isolates from blueberry tested in this study. Correlation trend line and statistics are indicated.

Pearson correlation analysis (**Figure 8**) indicated that there was a statistically significant positive correlation between EC_50_ values of boscalid and pydiflumetofen (r=0.52, p<0.05). Of the 12 isolates identified as having a reduced sensitivity to pydiflumetofen in this study, 11 (92%) were also identified as being resistant to boscalid (**Figures 5A, B**). These included all nine isolates determined to have EC_50_ values greater than 0.150 µg/mL for pydiflumetofen.

Of the 16 isolates for which succinate dehydrogenase gene sequences were obtained in this study, all seven isolates with an EC_50_ of greater than 0.092 µg/mL for pydiflumetofen had detectable mutations in at least one succinate dehydrogenase gene (**Table 5**). These seven isolates were all resistant to boscalid. For isolates with EC_50_ values for pydiflumetofen less than 0.092 µg/mL, only 2 of 9 isolates had a detectable mutation in a succinate dehydrogenase gene, and both of those isolates were resistant to boscalid (**Table 5**).

**Table 5:** SDHI fungicide sensitivity and mutation status of select *Alternaria* spp. isolates.

Isolate Name	Site Number	Boscalid Resistance Status	Mutations (sdh genes)	EC_50_ (µg/mL)	
				Boscalid	Pydiflumetofen
MB21-347	5	Sensitive	no mutations	1.053	0.018
MB21-358	6	Sensitive	no mutations	1.094	0.057
MB21-362	2	Sensitive	no mutations	0.784	0.029
MB21-363	5	Sensitive	no mutations	1.657	0.026
MB21-449	5	Resistant	no mutations	8.154	0.020
MB21-456	12	Resistant	no mutations	>100	0.023
MB21-479	5	Resistant	no mutations	>100	0.008
*MB21-068	5	Resistant	H134Q (sdhC)	>100	0.054
MB21-405	5	Resistant	H134Q (sdhC)	>100	0.377
*MB21-433	5	Resistant	H134Q (sdhC)	>100	0.093
*MB21-495	14	Resistant	H133R (sdhD)	>100	0.822
*MB21-500	14	Resistant	H133R (sdhD)	36.180	0.253
MB21-543	14	Resistant	H133R (sdhD)	13.703	0.026
MB21-544	14	Resistant	H133R (sdhD)	>100	1.114
*MB21-545	14	Resistant	H133R (sdhD)	26.720	0.359
*MB21-777	7	Resistant	G79R (sdhC)	>100	0.260

*indicates isolate with resistance to both pyraclostrobin and boscalid.

## 4 Discussion

Georgia routinely ranks amongst the top producers of blueberries in the U.S (NASS, 2022), but fruit rot diseases cause significant yield losses in the state each year. Fungicides are routinely applied in Georgia to manage blueberry fruit rot pathogens, including *Alternaria* spp. (Sial et al., 2023; Neugebauer et al., 2024). However, relatively little work had been previously done with *Alternaria* spp. from Georgia (Kaur and Dutta, 2024), and prior to the work described here there was no comprehensive data available regarding either the identities of or fungicide resistance status of *Alternaria* spp. isolates causing fruit rot in Georgia blueberries. As such, we identified the species of *Alternaria* isolates associated with fruit rot in Georgia blueberries and evaluated the resistance status of the obtained fungal isolates versus commonly used fungicides. Specifically, we determined the EC_50_ values of *Alternaria* spp. isolates for seven important fungicides that are currently used to manage pre- and post-harvest fruit rot diseases of blueberry in Georgia. These EC_50_ values give us the first detailed picture of the current fungicide sensitivity of *Alternaria* spp. isolates from blueberry in Georgia.

There are many different species of *Alternaria* that cause postharvest diseases in fruit crops; however, it is generally recognized that *A. alternata, A. tenuissima*, and *A. arborescens* are the primary species that cause Alternaria rot in blueberries (Neugebauer et al., 2024). In our study, we identified *A. alternata* to be the most abundant (93.5%) amongst the *Alternaria* spp. isolates cultured from blueberry fruit in Georgia. This finding is in agreement with previous work with *Alternaria* from blueberries in California, which found that the majority of isolates (61.5%) belonged to *A. alternata* (Zhu and Xiao, 2015). The remainder of isolates in our study were found to belong to other species including *A. tenuissima*, *A. limoniasperae*, and *A. dumosa* (1 isolate of each). Though the older literature describes *A. tenuissima* as the cause of Alternaria fruit rot (Milholland and Jones, 1972; Cline, 1996; Milholland and Cline, 2017), finding this species in low abundance is in agreement with the aforementioned study of blueberries in California which found that only 5% of isolates were *A. tenuissima*. Though *A. limoniasperae* and *A. dumosa* were not found by Zhu and Xiao (2015), based on the results of our pathogenicity testing of a selection of our *Alternaria* isolates, these two isolates were capable of rotting detached blueberry fruit, albeit with lower severities than all of the 19 A*. alternata* isolates and one *A. tenuissima* isolate we assayed. *A. dumosa* was recently reported to cause blueberry fruit rot in China (Wang et al., 2024a), but to our knowledge *A. limoniasporae* has not been previously reported as a cause of fruit rot on blueberries. Given these facts, and their low abundance among our collected isolates, these two species seem likely to be of less importance than *A. alternata* in causing Alternaria fruit rot on blueberry in Georgia.

*Alternaria* isolates with resistance to pyraclostrobin and boscalid, as well as isolates with reduced sensitivity to pydiflumetofen, were identified in our study, and all tested isolates were determined to be sensitive to the other four fungicides examined: fluazinam, metconazole, fludioxonil, and cyprodinil. For these fungicides, EC_50_ values were low or very low for all isolates and generally fell within ranges observed for fungicide-sensitive *Alternaria* isolates from blueberries or other crop systems (Mitani et al., 1996; Avenot and Michailides, 2015; Fonseka and Gudmestad, 2016; Gama et al., 2021; Haque and Parvin, 2022; Wang et al., 2022).

Fluazinam inhibits the development of appressoria and penetrating hyphae. For fluazinam, 39 of the isolates examined in our study had EC_50_ values less than 0.1 µg/mL, with the values for the remaining seven isolates falling between 0.1 to 0.2 µg/mL. This range is somewhat higher than, but comparable to, the values observed from prior studies of *A. alternata* isolates from sugar beet in the U.S. (0.0004 to 0.0021 µg/mL) and pear in Japan (less than 0.1 µg/mL) (Mitani et al., 1996; Haque and Parvin, 2022). Resistance to fluazinam is not frequently reported; however, resistance has been found in *Phytophthora infestans* on potato where it had been extensively used (Schepers et al., 2018). In recent years, fluazinam has been found to be very effective against *Colletotrichum* spp. that cause anthracnose fruit rot of blueberry, and a study of 201 C*. gloeosporioides* isolates collected from the blueberries in Florida indicated no resistance to fluazinam when isolates were screened at a discriminatory dose of 1 µg/mL (Gama et al., 2021). Though fluazinam has not been widely used in Georgia blueberry production previously, given the recent identification of pyraclostrobin and boscalid resistant *C. gloeosporioides* in Georgia blueberry (Ali et al., 2019), this effective fungicide has been recently recommended as part of a rotation to control QoI fungicide-resistant *Colletotrichum* on blueberry. Accordingly, the assessment of *Alternaria* isolate sensitivity in our study is particularly timely as fungal exposure to fluazinam is likely to increase in the coming years.

Metconazole is a DMI fungicide used for its efficacy against multiple fungal diseases including those caused by *Alternaria* spp. (Kumazawa et al., 2000; Fonseka and Gudmestad, 2016; Lee et al., 2021). Metconazole inhibits fungal cell membrane development by preventing ergosterol biosynthesis leading to disruption of cell membrane function, leakage of cytoplasmic contents, and hyphal inhibition (Wang et al., 2024b). Previous reports of EC_50_ values for *A. alternata* in other crops are rare, and resistance among *Alternaria* species to metconazole does not appear to have been reported previously. There is no baseline sensitivity information for metconazole and *A. alternata* in Georgia blueberries, and in comparison to a previous baseline sensitivity study of *A. alternata* from potato (range 0.05 to 0.46 µg/mL; mean 0.26 µg/mL) (Fonseka and Gudmestad, 2016), the EC_50_ values observed in our study (range 0.125 to 5.729 µg/mL; mean 1.146 µg/mL) were relatively higher. Despite this, the isolates in our study were still concluded to be sensitive based upon their unimodal frequency distribution and the fact that the minimum inhibitory concentration (MIC) was less than 10 μg/mL for all tested isolates. Furthermore, the isolate with the highest EC_50_ in our study (5.729 μg/mL) had a resistance factor of less than 5 when compared to the mean EC_50_. Reduced sensitivity to metconazole has been reported, in *Colletotrichum truncatum* from peach, with a mean EC_50_ value of 16.6 μg/mL (Chen et al., 2016). The data collected in our study on metconazole sensitivity will be a valuable basis for comparison if shifts in sensitivity occur in *Alternaria* spp. from Georgia blueberries.

Cyprodinil, a broad-spectrum pyrimidinamine fungicide that is used to protect fruit plants, vines, cereals, and vegetables from a wide range of fungal pathogens (Ma and Ye, 1997), works through inhibition of the biosynthesis of methionine and other thionic amino acids of fungi (Masner et al., 1994). Resistance to cyprodinil has not been frequently reported among *Alternaria* spp. but has been reported from other fungal species including *Botrytis cinerea*, where 30% of the isolates from strawberries were found to be resistant (Fernandez-Ortuno et al., 2013). For cyprodinil, 22 of the isolates in our study had EC_50_ values less than 0.1 µg/mL, 15 isolates were between 0.1 and 0.2 µg/mL, and the remaining nine isolates were between 0.2 and 0.4 µg/mL. These values, which ranged from 0.02 to 0.40 µg/mL with a mean value of 0.13 µg/mL were relatively lower than prior reports from fungicide-sensitive *A. alternata* from blueberries in California (mean 0.465 µg/mL) and baseline sensitivities established for *A. alternata* isolates from pistachio in California (range 0.001 to 1.184 µg/mL; mean 0.214 µg/mL) (Avenot and Michailides, 2015; Wang et al., 2022). This suggests that *A. alternata* isolates from Georgia blueberry remain sensitive to cyprodinil at this time, with mean EC_50_ values from Georgia being only one-third and one-half the means from fungicide-sensitive isolates from California blueberries and California pistachios, respectively.

With respect to fludioxonil, the EC_50_ values for our isolates ranged from 0.037 to 0.234 µg/mL, and as such were relatively lower than the baseline sensitivities (range 0.010 to 4.875 µg/mL) established for isolates from pistachio in California between 1998 and 2003 (Avenot and Michailides, 2015). Likewise, the mean EC_50_ for our isolates (0.124 µg/mL) was relatively similar to the mean (0.078 µg/mL) from a recent study of *A. alternata* isolates from blueberry in California which found, as we did, that all tested isolates were sensitive to fludioxonil (Wang et al., 2022). While there is no baseline sensitivity information available for fludioxonil and *Alternaria* spp. from blueberry or any other crops in Georgia, we anticipate that the EC_50_ values determined in our study will be valuable for future resistance monitoring efforts in Georgia, as fludioxonil is widely used in conventional blueberry production in Georgia, typically as one component of combination products with cyprodinil in the commercial product Switch^®^ (Syngenta Crop Protection, 2022) or with pydiflumetofen in the commercial product Miravis Prime^®^ (Syngenta Crop Protection, 2023). While resistance to fludioxonil has been reported in *Alternaria* spp. from pistachio and crucifers (Iacomi-Vasilescu et al., 2004; Avenot and Michailides, 2015), significant fitness costs associated with fludioxonil resistance that have been observed in the laboratory with isolates of other fludioxonil-resistant fungal species (Li and Xiao, 2008) may tend to slow the development of widespread fludioxonil resistance in some cases despite repeated applications.

The fungicide boscalid has been utilized in blueberry production in the U.S. since 2003, typically as one component of a two-component mixture with pyraclostrobin in the commercial product Pristine^®^ (BASF, 2003). Since that time, it has been widely used by Georgia blueberry growers to manage several diseases including fruit rots such as Alternaria leaf spot and fruit rot (*Alternaria* spp.). anthracnose ripe rot (*Colletotrichum* spp.), and Botrytis gray mold (*Botrytis cinerea*) (Sial et al., 2023). Perhaps not unexpectedly, given its long history of widespread use, we identified resistance to boscalid in 21 of 46 (46%) *Alternaria* isolates in our study. Moreover, among these isolates, 14 of 46 (30%) were extremely resistant to boscalid with EC_50_ values greater than 100 µg/mL. These findings are similar to findings from blueberry *A. alternata* from California, where 33% of isolates were reported to have EC_50_ values of greater than 100 µg/mL (Wang et al., 2022). By contrast, in comparison to Wang et al. (2022) where only 23% of isolates had EC_50_ values less than 10 µg/mL, our study indicated a larger proportion of isolates remaining sensitive to boscalid with 25 isolates (54%) having EC_50_ values less than 5 µg/mL. While there is no prior data regarding boscalid-resistant *Alternaria* spp. in Georgia in any crops, nor any baseline EC_50_ values for *Alternaria* spp. in Georgia to compare with, our data overall suggest that a shift has likely taken place (or is in progress) among *Alternaria* isolates from blueberry towards boscalid-resistance due to selection pressure from boscalid applications over the past two decades. This is not surprising based on observations from other crop systems. For example, prior to the introduction and use of boscalid, the baseline sensitivity to boscalid for 43 A*. alternata* isolates collected during 1999 and 2000 from pistachio in California showed that no resistant isolates were present and that EC_50_ values ranged from 0.011 to 0.650 µg/mL (Avenot et al., 2014). However, just a decade later, 69 of 117 (59%) *A. alternata* isolates collected from pistachio orchards in the Central Valley of California were found to be extremely resistant to boscalid with EC_50_ values greater than 100 µg/mL (Avenot and Michailides, 2015). In Georgia, while 21 isolates with boscalid resistance were identified in our study, these isolates originated from only 6 of 16 unique locations (with 16 out of 21 resistant isolates originating from only two of these locations) suggesting that resistance may not yet be widespread at this time. Continued resistance monitoring will be necessary, and the EC_50_ values for boscalid determined in our study will be valuable for this effort going forward.

Resistance to the SDHI fungicide boscalid has been previously associated with mutations within the genes encoding subunits *sdhB, sdhC*, or *sdhD* of the succinate-dehydrogenase complex (Sierotzki et al., 2011; Avenot et al., 2014). One of the most common mutations for *sdhB* in *A. alternata* is H277Y/R (Avenot et al., 2008a), however, in our analysis of boscalid-resistant isolates this mutation was not found in any tested *Alternaria* isolates from Georgia blueberry. Nonetheless, two other common mutations previously reported in *Alternaria* spp. (Avenot et al., 2009; Metz et al., 2019), corresponding to H134Q in *sdhC* and H133R in *sdhD*, were found in some of our boscalid-resistant isolates. Furthermore, G79R, a less-frequently reported mutation in *Alternaria* spp. (Förster et al., 2022), was also identified within *sdhC* of a single boscalid-resistant isolate in our study. Of note, all five isolates identified with the H133R mutation originated from a common location (site 14) and all three isolates identified with the H134Q mutation originated from a different common location (site 5) while the isolate with the G79R mutation was identified from a different site from the others, suggesting that resistance to boscalid in these locations likely developed independently. In addition, at least four isolates identified as having resistance to boscalid in our study (including some boscalid-resistant isolates from site 5 where the H134Q mutation was identified) did not have any detectable mutations within *sdhB*, *sdhC*, or *sdhD*. This is in agreement with prior reports suggesting that other determinants besides identifiable mutations in the succinate dehydrogenase subunit genes may play a role in resistance expression (Avenot et al., 2014; Förster et al., 2022).

Given the large number of isolates identified in this study with resistance to boscalid, and reports of cross-resistance among SDHI fungicides in some fungal pathogens (Avenot et al., 2014; Fernandez-Ortuno et al., 2017; Alzohairy et al., 2023), we also investigated the sensitivity of isolates in our study to the SDHI fungicide pydiflumetofen. Pydiflumetofen has recently begun to be utilized for managing blueberry fruit rots in Georgia and is most commonly applied in combination with fludioxonil in the commercial product Miravis Prime^®^ (Sial et al., 2023). For *Alternaria* isolates in our study, EC_50_ values for pydiflumetofen ranged from 0.008 to 1.114 µg/mL, which were somewhat higher than were found in a prior study conducted on *A*. *alternata* isolates causing Alternaria leaf spot of almond in California (range 0.001 to 0.215 µg/mL) (Förster et al., 2022), but more similar to results from *A*. *alternata* isolates causing black spot disease on cherry in China (range 0.027 to 1.175 µg/mL) (Siling et al., 2023). However, in contrast to the conclusions of Siling et al. (2023), where isolates were characterized as being sensitive to pydiflumetofen based on a unimodal distribution of EC_50_ values and the fact that they possessed no identifiable mutations within the succinate dehydrogenase subunit genes, the frequency distribution of our isolates’ EC_50_ values was bimodal and several isolates did possess mutations in *sdhC* or *sdhD*. From our analysis, 12 of our isolates showed reduced sensitivity to pydiflumetofen. Among these, 11 were resistant to boscalid, and there was a statistically significant positive correlation between the sensitivity of isolates in our study to these two SDHI fungicides. Of note, despite the fact that cross-resistance between different SDHI fungicides is assumed (FRAC, 2024a) and has been identified in varying degrees in pathogens such as *A. alternata* for some SDHI fungicide combinations (Avenot et al., 2014), our results stand in contrast to recent prior work with *A. alternata* which did not find strong evidence for cross-resistance among several SDHI fungicide combinations including boscalid and pydiflumetofen (Förster et al., 2022). Nonetheless, the observed correlation in our study, along with the fact that significant numbers of isolates showed reduced sensitivity to pydiflumetofen, may have significant implications for the long-term efficacy of products containing pydiflumetofen for Alternaria fruit rot control in Georgia, and these results suggest that additional fungicide resistance monitoring will be necessary going forward to stay abreast of potential shifts of isolates toward resistance to pydiflumetofen.

In addition to finding resistance to boscalid, 10 of 46 (22%) *Alternaria* isolates from this study were identified with resistance to the QoI fungicide pyraclostrobin. Pyraclostrobin is typically applied to blueberries in Georgia in the form of the commercial product Pristine^®^ (Sial et al., 2023), which includes boscalid as its other component. However, the singular use of other QoI fungicides, such as azoxystrobin, in blueberry production has a longer history. Given the numerous reports worldwide of resistance to QoI in multiple pathogen species (Fisher and Meunier, 2008), these fungicides are generally considered high risk for resistance development (FRAC, 2024b), and our identification of pyraclostrobin-resistant *Alternaria* isolates is, perhaps, not surprising. As with boscalid, there are no baseline pyraclostrobin EC_50_ values for *Alternaria* spp. from blueberry in Georgia; however, a previous baseline for *A. alternata* causing late blight of pistachios in California was developed using isolates collected from orchards without a previous history of Pristine^®^ applications (Avenot et al., 2008b). In Avenot et al. (2008b) most isolates (77%) were sensitive to pyraclostrobin with EC_50_ values less than 0.01 µg/mL, 17% had low resistance (mean EC_50_ value = 4.71 µg/mL), and a single isolate was resistant with an EC_50_ value greater than 100 µg/mL. That study, which like ours used a spore germination assay to assess *Alternaria* sensitivity to the strong spore germination inhibitory ability of QoI fungicides (Barilli et al., 2016), set a cutoff between sensitive and resistant isolates of 10 µg/mL (Avenot et al., 2008b). Based on this cutoff, out of 46 *Alternaria* spp. isolates in our study, 10 were found to be resistant and 36 sensitive to pyraclostrobin, and all 10 resistant isolates were confirmed to possess the G143A mutation frequently identified in QoI-resistant fungal pathogens (Fisher and Meunier, 2008). Among the 36 sensitive isolates, 28 (77%) had EC_50_ values less than 1 µg/mL. By contrast, 3 of 10 resistant isolates in our study were extremely resistant (EC_50_ values greater than 100 µg/mL), with the remaining seven resistant isolates having a mean EC_50_ value of 38.98 µg/mL. While a significant number of resistant isolates were found in our study, the proportion of resistant isolates is low in comparison to previous reports from pistachio and blueberry fields in California with a history of Pristine^®^ applications, where 95% and 42%, respectively, were determined to be resistant to pyraclostrobin (Avenot et al., 2008b; Wang et al., 2022)

In our study, 6 of 46 (13%) isolates were resistant to both boscalid and pyraclostrobin, and five of these isolates showed reduced sensitivity to pydiflumetofen. This indicates that multiple fungicide resistance (including resistance to both components of Pristine^®^) is present among *Alternaria* isolates from Georgia blueberry. Multiple resistance to both components of Pristine^®^ has been reported before in *Alternaria* species, including in pistachio orchards where 7 of 59 isolates (12%) were found to be resistant to boscalid and pyraclostrobin (Avenot et al., 2008b), and in Georgia blueberries, *Colletotrichum gloeosporioides* isolates causing anthracnose ripe rot were recently found to be resistant to both of these fungicides as well (Ali et al., 2019). The presence of multiple fungicide resistance in Georgia blueberries has the potential to significantly reduce the efficacy of spray programs currently being utilized to control fruit rots. The extent and prevalence of multiple fungicide resistance should be monitored in the future, and growers should be encouraged to use tank mixes with other modes of action and multisite fungicides before control failures occur.

Taken together, the identification of the primary species associated with Alternaria fruit rot on Georgia blueberries, the characterization of fungicide sensitivity of *Alternaria* isolates, and the identification of fungicide resistance and fungicide resistance-associated mutations will aid in the management of this fruit rot disease in Georgia. Informed decisions regarding spray selection as well as more accurate identification and diagnosis of this issue are expected to result from this work.

## Data Availability

The datasets presented in this study can be found in online repositories. The names of the repository/repositories and accession number(s) can be found in the article/**Supplementary Material**.

## Supplementary material

Supplementary Figure 1 Collection locations for *Alternaria* spp. isolates utilized in this study. Counties within Georgia (U.S.A.) where isolates were collected are shown in red and numbers of isolates collected in each respective county are indicated in parentheses.

Supplementary Figure 2 Disease index scale utilized in pathogenicity tests. On the scale: 0 = no fungal growth; 1 = growth restricted within the stem scar site (less than 4 mm growth); 2 = growth covering the entire stem scar site (~4 mm); 3 = growth beyond the stem scar site (>4 mm to 7 mm); 4 = growth covering most of the berry surface, and 5 = mushy berry with growth across the entire berry surface.
