# Supplementary figures and images for: Fungicide resistance profiles of Alternaria spp. associated with fruit rot of blueberry in Georgia, USA
Source: Front Plant Sci. 2025 Feb 27;16:1524586. doi: 10.3389/fpls.2025.1524586 (PMC11903456; doi:10.3389/fpls.2025.1524586)

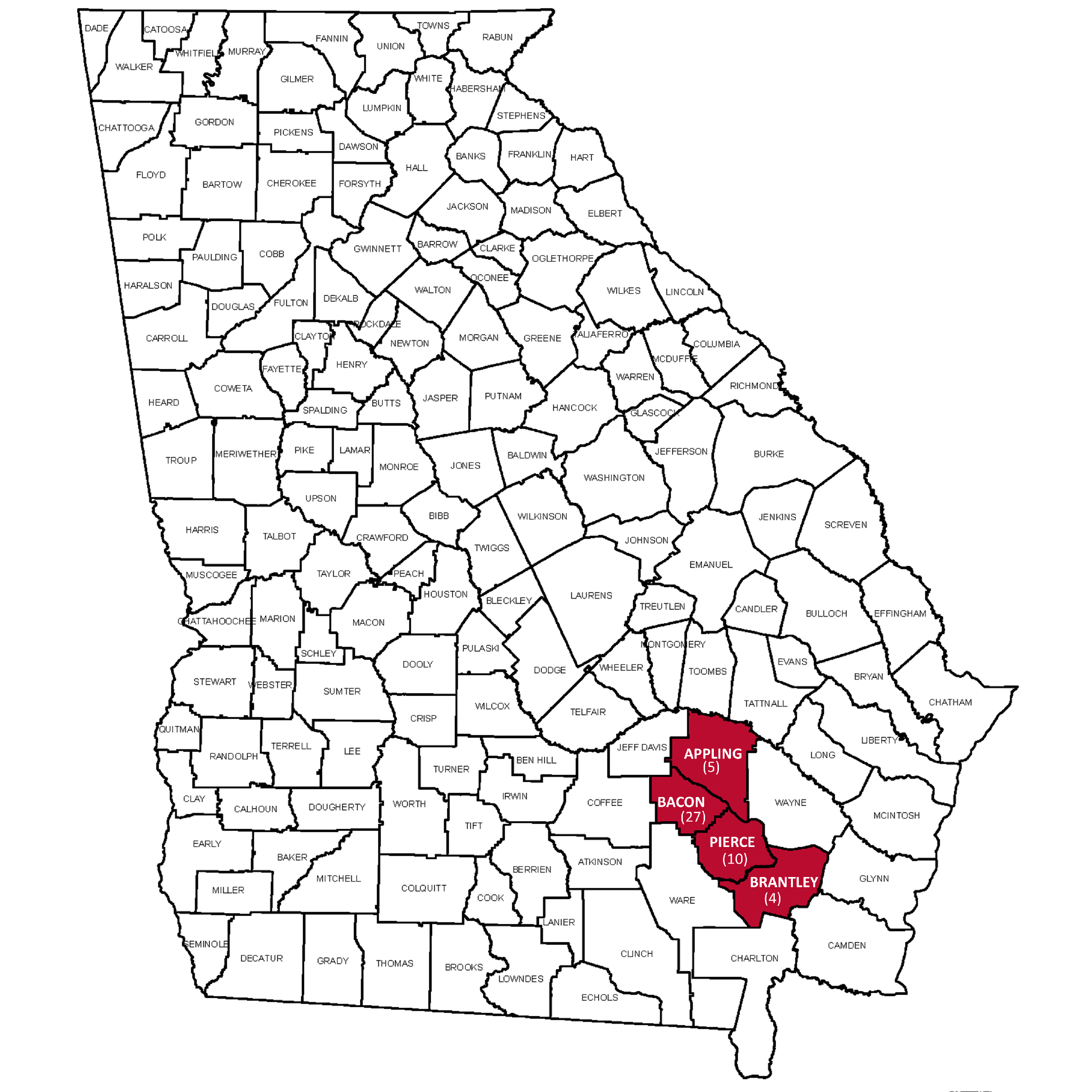

Supplement: Supplementary Figure 1 — Collection locations for Alternaria spp. isolates utilized in this study. Counties within Georgia (U.S.A.) where isolates were collected are shown in red and numbers of isolates collected in each respective county are indicated in parentheses. [file Image1.tif]

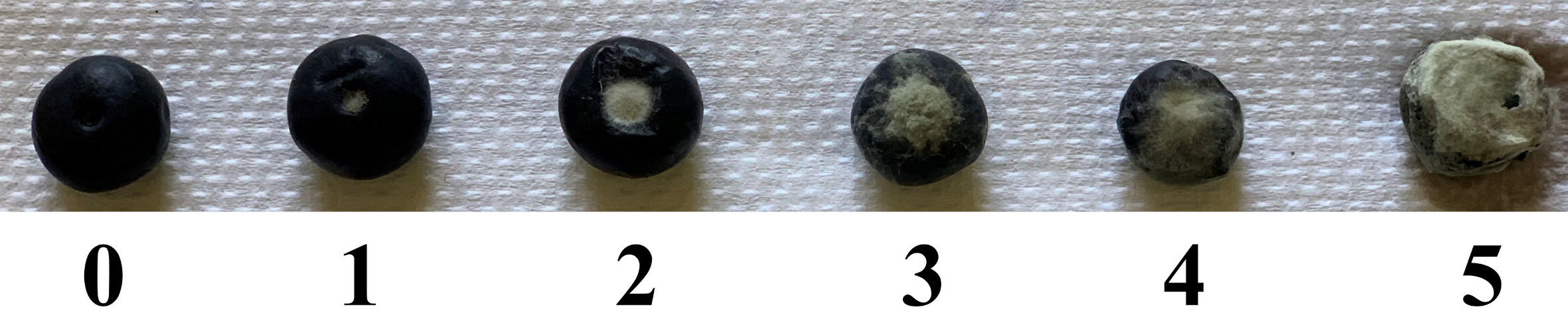

Supplement: Supplementary Figure 2 — Disease index scale utilized in pathogenicity tests. On the scale: 0 = no fungal growth; 1 = growth restricted within the stem scar site (less than 4 mm growth); 2 = growth covering the entire stem scar site (~4 mm); 3 = growth beyond the stem scar site (>4 mm to 7 mm); 4 = growth covering most of the berry surface, and 5 = mushy berry with growth across the entire berry surface. [file Image2.tif]
