# Supplementary material for: Fungicide resistance profiles of Alternaria spp. associated with fruit rot of blueberry in Georgia, USA
Source: Front Plant Sci. 2025 Feb 27;16:1524586. doi: 10.3389/fpls.2025.1524586 (PMC11903456; doi:10.3389/fpls.2025.1524586)
Supplement: Supplementary file 3 [file Table1.docx]

**Supplementary Table 1.** Accession numbers of additional sequences utilized in phylogenetic analysis.

| Isolate Name | Species | Reference | Accession Numbers | | | |
| --- | --- | --- | --- | --- | --- | --- |
|  |  |  | ATPase | Alt a1 | CAL | RPB2 |
| CBS 577.94 | *Alternaria agripestis* | Lawrence et al. 2013 | JQ671932 | JQ646440 | JQ646266 | JQ646468 |
| CBS 118809 | *Alternaria alstroemeriae* | Elfar et al. 2023 | MH101803 | MH084526 | MH175185 | n/a |
| EGS 52-039 | *Alternaria alternantherae* | Lawrence et al. 2013 | JQ671892 | JN383511 | JQ646226 | n/a |
| EGS 34-016 | *Alternaria alternata* | Lawrence et al. 2013 | JQ671874 | AY563301 | JQ646208 | JQ646490 |
| X1122 | *Alternaria alternata* | Zhu and Xiao 2015 | KJ908253 | KJ920989 | KJ920970 | n/a |
| X1265 | *Alternaria alternata* | Zhu and Xiao 2015 | KJ908237 | KJ921004 | KJ920960 | n/a |
| CBS 594.93 | *Alternaria aragakii* | Lawrence et al. 2013 | JQ671935 | JQ646443 | JQ646269 | JQ646521 |
| EGS 39-128 | *Alternaria arborescens* | Lawrence et al. 2013 | JQ671880 | AY563303 | JQ646214 | JQ646487 |
| X1033 | *Alternaria arborescens* | Zhu and Xiao 2015 | KJ908248 | KJ921009 | KJ920941 | n/a |
| X1263 | *Alternaria arborescens* | Zhu and Xiao 2015 | KJ908226 | KJ921003 | KJ920947 | n/a |
| EGS 35-122 | *Alternaria argyroxiphii* | Lawrence et al. 2013 | JQ671926 | JQ646434 | JQ646260 | JQ646464 |
| CBS 118810 | *Alternaria betae-kenyensis* | Elfar et al. 2023 | MH101805 | JQ905104 | MH175189 | n/a |
| DAOM 231361 | *Alternaria bornmuelleri* | Lawrence et al. 2013 | JQ671791 | JN383516 | JQ646125 | JQ646520 |
| ATCC 18043 | *Alternaria botrytis* | Lawrence et al. 2013 | JQ671834 | AY563317 | JQ646168 | n/a |
| EEB 2232 | *Alternaria brassicicola* | Lawrence et al. 2013 | JQ671843 | AY563311 | JQ646177 | n/a |
| CBS 107.38 | *Alternaria burnsii* | Lawrence et al. 2013 | JQ671860 | JQ646388 | JQ646194 | JQ646457 |
| EGS 26-010 | *Alternaria carotiincultae* | Lawrence et al. 2013 | JQ671850 | AY563287 | JQ646184 | JQ646477 |
| ATCC 18044 | *Alternaria chartarum* | Lawrence et al. 2013 | JQ671828 | AY563319 | JQ646162 | n/a |
| EGS 41-188 | *Alternaria cheiranthi* | Lawrence et al. 2013 | JQ671830 | AY563290 | JQ646164 | n/a |
| CBS 109164 | *Alternaria cretica* | Lawrence et al. 2013 | JQ671916 | JQ646426 | JQ646250 | JQ646480 |
| EGS 31-021 | *Alternaria cucurbitae* | Lawrence et al. 2013 | JQ671836 | AY563315 | JQ646170 | JQ646452 |
| CBS 915.96 | *Alternaria dianthicola* | Lawrence et al. 2013 | JQ671810 | n/a | JQ646144 | JQ646465 |
| EGS 45-007 | *Alternaria dumosa* | Lawrence et al. 2013 | JQ671877 | AY563305 | JQ646211 | n/a |
| CBS 489.92 | *Alternaria eichhorniae* | Woudenberg et al. 2015 | MH101806 | KP123973 | MH175190 | n/a |
| EGS 38-073 | *Alternaria embellisia* | Lawrence et al. 2013 | JQ671793 | AY563322 | JQ646127 | n/a |
| EGS 37-143 | *Alternaria ethzedia* | Lawrence et al. 2013 | JQ671805 | AY563284 | JQ646139 | JQ646458 |
| EGS 42-049 | *Alternaria euphorbiicola* | Lawrence et al. 2013 | JQ671911 | AY563314 | JQ646245 | n/a |
| EGS 36-103 | *Alternaria eureka* | Lawrence et al. 2013 | JQ671771 | JN383507 | JQ646105 | JQ646473 |
| EGS 44-001 | *Alternaria frumenti* | Lawrence et al. 2013 | JQ671823 | JQ646378 | JQ646157 | JQ646509 |
| 0407-5-2 | *Alternaria gaisen* | Yan et al. 2024 | MW541812 | MW541814 | n/a | n/a |
| CBS 104.32 | *Alternaria gossypina* | Lawrence et al. 2013 | JQ671868 | JQ646395 | JQ646202 | n/a |
| CBS 107.41 | *Alternaria gypsophilae* | Lawrence et al. 2013 | JQ671859 | JQ646387 | JQ646193 | JQ646475 |
| EGS 50-184 | *Alternaria hordeicola* | Lawrence et al. 2013 | JQ671812 | JQ646372 | JQ646146 | n/a |
| EGS 49-062 | *Alternaria hyacinthi* | Lawrence et al. 2013 | JQ671778 | FJ266506 | JQ646112 | n/a |
| EGS 27-193 | *Alternaria infectoria* | Lawrence et al. 2013 | JQ671804 | FJ266502 | JQ646138 | n/a |
| CBS 118486 | *Alternaria iridiaustralis* | Woudenberg et al. 2015 | MH101807 | KP123981 | MH175191 | n/a |
| YZU 171616 | *Alternaria jacinthicola* | Luo et al. 2018 | MG781017 | MG781013 | MG781016 | n/a |
| EGS 41-158 | *Alternaria japonica* | Lawrence et al. 2013 | JQ671840 | AY563312 | JQ646174 | n/a |
| EGS 40-187 | *Alternaria leptinellae* | Lawrence et al. 2013 | JQ671773 | JQ646366 | JQ646107 | JQ646461 |
| C.FR13 | *Alternaria limoniasperae* | Elfar et al. 2019 | MH492694 | n/a | n/a | n/a |
| EGS 45-100 | *Alternaria limoniasperae* | Lawrence et al. 2013 | JQ671879 | AY563306 | JQ646213 | n/a |
| EGS 30-033 | *Alternaria longipes* | Lawrence et al. 2013 | JQ671864 | AY563304 | JQ646198 | JQ646515 |
| CBS 135.31 | *Alternaria malorum* | Lawrence et al. 2013 | JQ671800 | JQ646369 | JQ646134 | JQ646481 |
| EGS 31-061 | *Alternaria mouchaccae* | Lawrence et al. 2013 | JQ671799 | AY563279 | JQ646133 | JQ646459 |
| RC O1B9 | *Alternaria oxytropis* | Lawrence et al. 2013 | JQ671792 | JN383517 | JQ646126 | JQ646479 |
| EGS 29-180 | *Alternaria panax* | Lawrence et al. 2013 | JQ671846 | JQ646382 | JQ646180 | n/a |
| P354.8 | *Alternaria penicillata* | Lawrence et al. 2013 | JQ671788 | JN383502 | JQ646122 | JQ646469 |
| EGS 09-159 | *Alternaria petroselini* | Lawrence et al. 2013 | JQ671854 | AY563288 | JQ646188 | JQ646474 |
| EGS 41-130 | *Alternaria rosae* | Lawrence et al. 2013 | JQ671803 | JQ646370 | JQ646137 | n/a |
| EGS 19-016 | *Alternaria scirpicola* | Lawrence et al. 2013 | JQ671781 | AY563320 | JQ646115 | JQ646455 |
| ATCC 58177 | *Alternaria solani* | Lawrence et al. 2013 | JQ671898 | AY563299 | JQ646232 | JQ646507 |
| EGS 46-051 | *Alternaria sonchi* | Lawrence et al. 2013 | JQ671849 | AY563307 | JQ646183 | n/a |
| EGS 34-015 | *Alternaria tenuissima* | Lawrence et al. 2013 | JQ671875 | AY563302 | JQ646209 | n/a |
| SY-4 | *Alternaria tenuissima* | Qian et al. 2022 | MT416124 | MK593137 | MT416125 | n/a |
| CBS 114.35 | *Alternaria tomato* | Lawrence et al. 2013 | JQ671861 | JQ646389 | JQ646195 | JQ646494 |
| CBS 116533 | *Alternaria vaccariae* | Lawrence et al. 2013 | JQ671858 | JQ646386 | JQ646192 | JQ646463 |
