# Supplementary material for: Fungicide resistance profiles of Alternaria spp. associated with fruit rot of blueberry in Georgia, USA
Source: Front Plant Sci. 2025 Feb 27;16:1524586. doi: 10.3389/fpls.2025.1524586 (PMC11903456; doi:10.3389/fpls.2025.1524586)
Supplement: Supplementary file 4 [file Table2.docx]

**Supplementary Table 2.** Accession numbers for sequences generated from isolates utilized in this study.

| Isolate Name | Collection Date | Isolation Location  (Georgia, USA) | Site ID | Blueberry Type  (Cultivar) |  | Accession Number | | | | | | | | | | | | | | | | |  |
| --- | --- | --- | --- | --- | --- | --- | --- | --- | --- | --- | --- | --- | --- | --- | --- | --- | --- | --- | --- | --- | --- | --- | --- |
|  |  |  |  |  | ITS | | | ATPase | | Alt a1 | | CAL | | RPB2 | | cytB | | sdhB | | sdhC | | sdhD | |
| MB21-007 | 22-Apr-21 | Brantley County | Site 11 | SHB (Optimus) | OR041698 | | | OR091105 | | n/a | | n/a | | n/a | | OR091092 | | n/a | | n/a | | n/a | |
| MB21-008 | 22-Apr-21 | Brantley County | Site 11 | SHB (Optimus) | OR041699 | | | OR091106 | | n/a | | n/a | | n/a | | OR091093 | | n/a | | n/a | | n/a | |
| MB21-013 | 22-Apr-21 | Brantley County | Site 9 | SHB (V1) | OR041700 | | | OR091107 | | n/a | | n/a | | n/a | | OR091094 | | n/a | | n/a | | n/a | |
| MB21-068 | 17-May-21 | Bacon County | Site 5 | SHB (Star) | OR041701 | | | OR091108 | | PP662487 | | PP662470 | | PP662476 | | OR091095 | | PP620128 | | PP620136 | | PP620144 | |
| MB21-099 | 4-May-21 | Appling County | Site 1 | SHB (Farthing) | OR041702 | | | OR091109 | | n/a | | n/a | | n/a | | OR091096 | | n/a | | n/a | | n/a | |
| MB21-131 | 17-May-21 | Bacon County | Site 5 | SHB (Star) | OR041703 | | | OR091110 | | n/a | | n/a | | n/a | | OR091097 | | n/a | | n/a | | n/a | |
| MB21-280 | 17-May-21 | Bacon County | Site 5 | SHB (Star) | OR041704 | | | OR091111 | | n/a | | n/a | | n/a | | n/a | | n/a | | n/a | | n/a | |
| MB21-341 | 17-May-21 | Bacon County | Site 3 | SHB (Farthing) | OR041705 | | | OR091112 | | n/a | | n/a | | n/a | | n/a | | n/a | | n/a | | n/a | |
| MB21-347 | 17-May-21 | Bacon County | Site 5 | SHB (Star) | OR041706 | | | OR091113 | | PP662488 | | n/a | | n/a | | n/a | | PP620129 | | PP620137 | | OR091081 | |
| MB21-348 | 4-May-21 | Appling County | Site 1 | SHB (Farthing) | OR041707 | | | OR091114 | | n/a | | n/a | | n/a | | n/a | | n/a | | n/a | | n/a | |
| MB21-358 | 17-May-21 | Bacon County | Site 6 | SHB (Indigocrisp) | OR041708 | | | OR091115 | | PP662489 | | n/a | | n/a | | n/a | | OR091065 | | OR091073 | | OR091082 | |
| MB21-362 | 4-May-21 | Appling County | Site 2 | SHB (Farthing) | OR041709 | | | OR091116 | | PP662490 | | PP662471 | | PP662477 | | OR091098 | | OR091066 | | OR091074 | | OR091083 | |
| MB21-363 | 7-May-21 | Bacon County | Site 5 | SHB (Star) | OR041710 | | | OR091117 | | PP662491 | | PP662472 | | PP662478 | | n/a | | PP620130 | | PP620138 | | OR091084 | |
| MB21-375 | 17-May-21 | Bacon County | Site 5 | SHB (Star) | OR041711 | | | OR091118 | | n/a | | n/a | | n/a | | n/a | | n/a | | n/a | | n/a | |
| MB21-376 | 17-May-21 | Bacon County | Site 3 | SHB (Farthing) | OR041712 | | | OR091119 | | n/a | | n/a | | n/a | | n/a | | n/a | | n/a | | n/a | |
| MB21-397 | 17-May-21 | Bacon County | Site 3 | SHB (Farthing) | OR041713 | | | OR091120 | | PP662492 | | n/a | | n/a | | n/a | | n/a | | n/a | | n/a | |
| MB21-400 | 4-May-21 | Appling County | Site 1 | SHB (Farthing) | OR041714 | | | OR091121 | | PP662493 | | n/a | | n/a | | n/a | | n/a | | n/a | | n/a | |
| MB21-402 | 4-May-21 | Brantley County | Site 10 | SHB (Patrecia) | OR041715 | | | OR091122 | | n/a | | n/a | | n/a | | OR091099 | | n/a | | n/a | | n/a | |
| MB21-405 | 17-May-21 | Bacon County | Site 5 | SHB (Star) | OR041716 | | | OR091123 | | PP662494 | | n/a | | n/a | | n/a | | PP620131 | | PP620139 | | OR091085 | |
| MB21-410 | 7-May-21 | Pierce County | Site 15 | SHB (Rebel) | OR041717 | | | OR091124 | | n/a | | n/a | | n/a | | n/a | | n/a | | n/a | | n/a | |
| MB21-416 | 17-May-21 | Bacon County | Site 3 | SHB (Farthing) | OR041718 | | | OR091125 | | n/a | | n/a | | n/a | | n/a | | n/a | | n/a | | n/a | |
| MB21-417 | 7-May-21 | Pierce County | Site 13 | SHB (Farthing) | OR041719 | | | OR091126 | | n/a | | n/a | | n/a | | n/a | | n/a | | n/a | | n/a | |
| MB21-420 | 17-May-21 | Bacon County | Site 5 | SHB (Star) | OR041720 | | | OR091127 | | n/a | | n/a | | n/a | | n/a | | n/a | | n/a | | n/a | |
| MB21-421 | 17-May-21 | Bacon County | Site 3 | SHB (Farthing) | OR041721 | | | OR091128 | | n/a | | n/a | | n/a | | n/a | | n/a | | n/a | | n/a | |
| MB21-427 | 4-May-21 | Appling County | Site 2 | SHB (Farthing) | OR041722 | | | OR091129 | | n/a | | n/a | | n/a | | n/a | | n/a | | n/a | | n/a | |
| MB21-428 | 17-May-21 | Bacon County | Site 5 | SHB (Star) | OR041723 | | | OR091130 | | PP662495 | | n/a | | n/a | | n/a | | n/a | | n/a | | n/a | |
| MB21-433 | 17-May-21 | Bacon County | Site 5 | SHB (Star) | OR041724 | | | OR091131 | | PP662496 | | n/a | | n/a | | OR091100 | | PP620132 | | PP620140 | | PP620145 | |
| MB21-449 | 17-May-21 | Bacon County | Site 5 | SHB (Star) | OR041725 | | | OR091132 | | PP662497 | | n/a | | n/a | | n/a | | OR091067 | | OR091075 | | OR091086 | |
| MB21-454 | 7-May-21 | Pierce County | Site 16 | SHB (Star) | OR041726 | | | OR091133 | | n/a | | n/a | | n/a | | n/a | | n/a | | n/a | | n/a | |
| MB21-456 | 1-May-21 | Pierce County | Site 12 | SHB (Suziblue) | OR041727 | | | OR091134 | | PP662498 | | PP662473 | | PP662479 | | n/a | | OR091068 | | OR091076 | | OR091087 | |
| MB21-474 | 7-May-21 | Pierce County | Site 13 | SHB (Farthing) | OR041728 | | | OR091135 | | n/a | | n/a | | n/a | | n/a | | n/a | | n/a | | n/a | |
| MB21-475 | 17-May-21 | Bacon County | Site 5 | SHB (Star) | OR041729 | | | OR091136 | | PP662499 | | PP662474 | | PP662480 | | n/a | | n/a | | n/a | | n/a | |
| MB21-479 | 17-May-21 | Bacon County | Site 5 | SHB (Star) | OR041730 | | | OR091137 | | PP662500 | | n/a | | n/a | | n/a | | OR091069 | | OR091077 | | OR091088 | |
| MB21-483 | 17-May-21 | Bacon County | Site 5 | SHB (Star) | OR041731 | | | OR091138 | | n/a | | n/a | | n/a | | n/a | | n/a | | n/a | | n/a | |
| MB21-495 | 7-May-21 | Pierce County | Site 14 | SHB (Meadowlark) | OR041732 | | | OR091139 | | PP662501 | | PP662475 | | PP662481 | | OR091101 | | OR091070 | | OR091078 | | OR091089 | |
| MB21-500 | 7-May-21 | Pierce County | Site 14 | SHB (Meadowlark) | OR041733 | | | OR091140 | | PP662502 | | n/a | | n/a | | OR091102 | | PP620133 | | PP620141 | | PP620146 | |
| MB21-543 | 7-May-21 | Pierce County | Site 14 | SHB (Meadowlark) | OR041734 | | | OR091141 | | PP662503 | | n/a | | n/a | | n/a | | OR091071 | | OR091079 | | OR091090 | |
| MB21-544 | 7-May-21 | Pierce County | Site 14 | SHB (Meadowlark) | OR041735 | | | OR091142 | | PP662504 | | n/a | | n/a | | n/a | | OR091072 | | OR091080 | | OR091091 | |
| MB21-545 | 7-May-21 | Pierce County | Site 14 | SHB (Meadowlark) | OR041736 | | | OR091143 | | PP662505 | | n/a | | n/a | | OR091103 | | PP620134 | | PP620142 | | PP620147 | |
| MB21-546 | 25-May-21 | Bacon County | Site 7 | SHB (Farthing) | OR041737 | | | OR091144 | | n/a | | n/a | | n/a | | n/a | | n/a | | n/a | | n/a | |
| MB21-561 | 17-May-21 | Bacon County | Site 7 | SHB (Farthing) | OR041738 | | | OR091145 | | n/a | | n/a | | n/a | | n/a | | n/a | | n/a | | n/a | |
| MB21-736 | 14-Jun-21 | Bacon County | Site 4 | RE (Brightwell) | OR041739 | | OR091146 | | n/a | | n/a | | n/a | | n/a | | n/a | | n/a | | n/a | | |
| MB21-754 | 14-Jun-21 | Bacon County | Site 8 | RE (Brightwell) | OR041740 | | OR091147 | | PP662506 | | n/a | | n/a | | n/a | | n/a | | n/a | | n/a | | |
| MB21-777 | 17-May-21 | Bacon County | Site 7 | SHB (Farthing) | OR041741 | | OR091148 | | PP662507 | | n/a | | n/a | | OR091104 | | PP620135 | | PP620143 | | PP620148 | | |
| MB21-778 | 17-May-21 | Bacon County | Site 7 | SHB (Farthing) | OR041742 | | OR091149 | | PP662508 | | n/a | | n/a | | n/a | | n/a | | n/a | | n/a | | |
| MB21-779 | 14-Jun-21 | Bacon County | Site 4 | RE (Brightwell) | OR041743 | | OR091150 | | n/a | | n/a | | n/a | | n/a | | n/a | | n/a | | n/a | | |
